# Differential excitatory control of 2 parallel basket cell networks in amygdala microcircuits

**DOI:** 10.1371/journal.pbio.2001421

**Published:** 2017-05-24

**Authors:** Tibor Andrási, Judit M. Veres, Laura Rovira-Esteban, Richárd Kozma, Attila Vikór, Erzsébet Gregori, Norbert Hájos

**Affiliations:** 1 Lendület Laboratory of Network Neurophysiology, Institute of Experimental Medicine, Hungarian Academy of Sciences, Budapest, Hungary; 2 János Szentágothai School of Neurosciences, Semmelweis University, Budapest, Hungary; ICM - Institut du Cerveau et de la Moelle épinière, France

## Abstract

Information processing in neural networks depends on the connectivity among excitatory and inhibitory neurons. The presence of parallel, distinctly controlled local circuits within a cortical network may ensure an effective and dynamic regulation of microcircuit function. By applying a combination of optogenetics, electrophysiological recordings, and high resolution microscopic techniques, we uncovered the organizing principles of synaptic communication between principal neurons and basket cells in the basal nucleus of the amygdala. In this cortical structure, known to be critical for emotional memory formation, we revealed the presence of 2 parallel basket cell networks expressing either parvalbumin or cholecystokinin. While the 2 basket cell types are mutually interconnected within their own category via synapses and gap junctions, they avoid innervating each other, but form synaptic contacts with axo-axonic cells. Importantly, both basket cell types have the similar potency to control principal neuron spiking, but they receive excitatory input from principal neurons with entirely diverse features. This distinct feedback synaptic excitation enables a markedly different recruitment of the 2 basket cell types upon the activation of local principal neurons. Our data suggest fundamentally different functions for the 2 parallel basket cell networks in circuit operations in the amygdala.

## Author summary

The perisomatic region of neurons refers collectively to the membrane surface of the cell body or soma, proximal dendrites, and axon initial segment. This is a unique functional domain in which the activity of a neuron can be controlled in the most effective manner. In the cerebral cortex, the perisomatic region of excitatory principal cells is solely innervated by inhibitory interneurons, which can be divided into 3 functional groups: axo-axonic cells and 2 types of basket cells. The reason why 3 distinct types of inhibitory cells are specialized to control principal cell firing is still unknown. To reveal the possible differences in the role of the 3 interneuron types played in cortical operation, we have investigated the organizing principles of synaptic communication between principal cells and inhibitory cell types in the basal nucleus of the amygdala. In this cortical structure, known to be critical for affective behavior, we revealed that the 2 basket cell types avoid innervating each other but contact axo-axonic cells. Both basket cell types have a similar potency to control principal cell firing, but they receive excitatory input from principal cells with entirely distinct features. Our data suggest fundamentally different functions for the 2 parallel basket cell networks in amygdala operation.

## Introduction

Gamma-aminobutyric acid (GABA)ergic basket and axo-axonic cells (AACs) targeting the perisomatic region of cortical principal neurons (PNs) are in a key position to effectively control the firing of their postsynaptic partners [1, 2]. Consequently, perisomatic inhibitory interneurons are essential for neural computations and thus cognitive processes like learning and memory, perception, and motor control [3, 4]. The critical role that these inhibitory interneurons play in circuit operation is reflected in the wide variety of neurological and psychiatric diseases that have been implicated in their malfunction, including epilepsy, schizophrenia, and autism [5–7].

In cortical structures, 2 distinct types of basket cells (BCs) expressing either a Ca^2+^ binding protein parvalbumin (PV) or a neuropeptide cholecystokinin (CCK) and cannabinoid receptor type 1 (CB1) give rise to the main inhibitory input onto the somata and proximal dendrites of excitatory PNs, while their axon initial segment is innervated by PV-expressing AACs (also referred as chandelier cells)[8–10]. The 2 BC types are markedly dissimilar in many single-cell features and are thought to generate postsynaptic inhibition with different properties [11–14]. In silico studies suggested that the synaptic connectivity among BCs is a key determinant of neuronal operation, especially during synchronous network activities [15]. Indeed, electrophysiological investigations provide evidence for a role of interconnected BC networks in the generation of gamma oscillations [16] and sharp wave-ripples [17, 18], rhythmic activities that are associated with memory acquisition and consolidation, respectively, in the hippocampus [19]. While previous work uncovered that cholecystokinin-expressing basket cells (CCKBCs) and parvalbumin-containing basket cells (PVBCs) innervate their own kind [20–24], it is still unclear whether the 2 BC types target each other, giving rise to a “super-network” of BCs that could very efficiently regulate spiking of their target neurons, primarily supervising the local circuit operation. Alternatively, they may form 2 parallel GABAergic networks without any synaptic cross-talk, a circuit organization that could substantially increase the adjustability and computational power in cortical networks [25]. In the latter case, the 2 BC types should receive distinct excitatory input, e.g., from local collaterals of PNs to fulfill independent operations.

To address these fundamental questions, we combined electrophysiological and neuroanatomical techniques with optogenetics in the basal amygdala (BA), a nucleus of the basolateral amygdala complex (BLA) that is known to be a site of plastic changes during fear learning [26–28]. This amygdala nucleus together with the lateral nucleus are viewed as nuclear extensions of the neocortex [29]. Although the PNs often with stellate-like appearance in the BA do not form a layered structure, many other features of these neurons match to neocortical pyramidal cells, including their excitatory nature, intrinsic membrane characteristics, connectivity patterns, and plastic properties [27, 30]. Moreover, the diversity of local GABAergic cells in the BA resembles that observed in neocortical structures [31, 32]. As in the neocortex or hippocampus, PNs in this amygdala nucleus are innervated by the 3 perisomatic inhibitory cell types [33–36], providing the structural basis for investigating the synaptic communication between BCs.

Our results uncovered the presence of 2 parallel BC networks in the BA. At the single-cell level, both BC types have the same potency to control the spiking of PNs that in turn distinctly excite these 2 interneuron types. Thus, PVBCs and CCKBCs in the BA form 2 separate GABAergic circuits whose activity is driven differentially by local excitatory neurons, which may be a general circuit organizing principle in cortical regions, contributing critically to local information processing.

## Results

### Two parallel BC circuits are present in the BA

**Fig 1:**
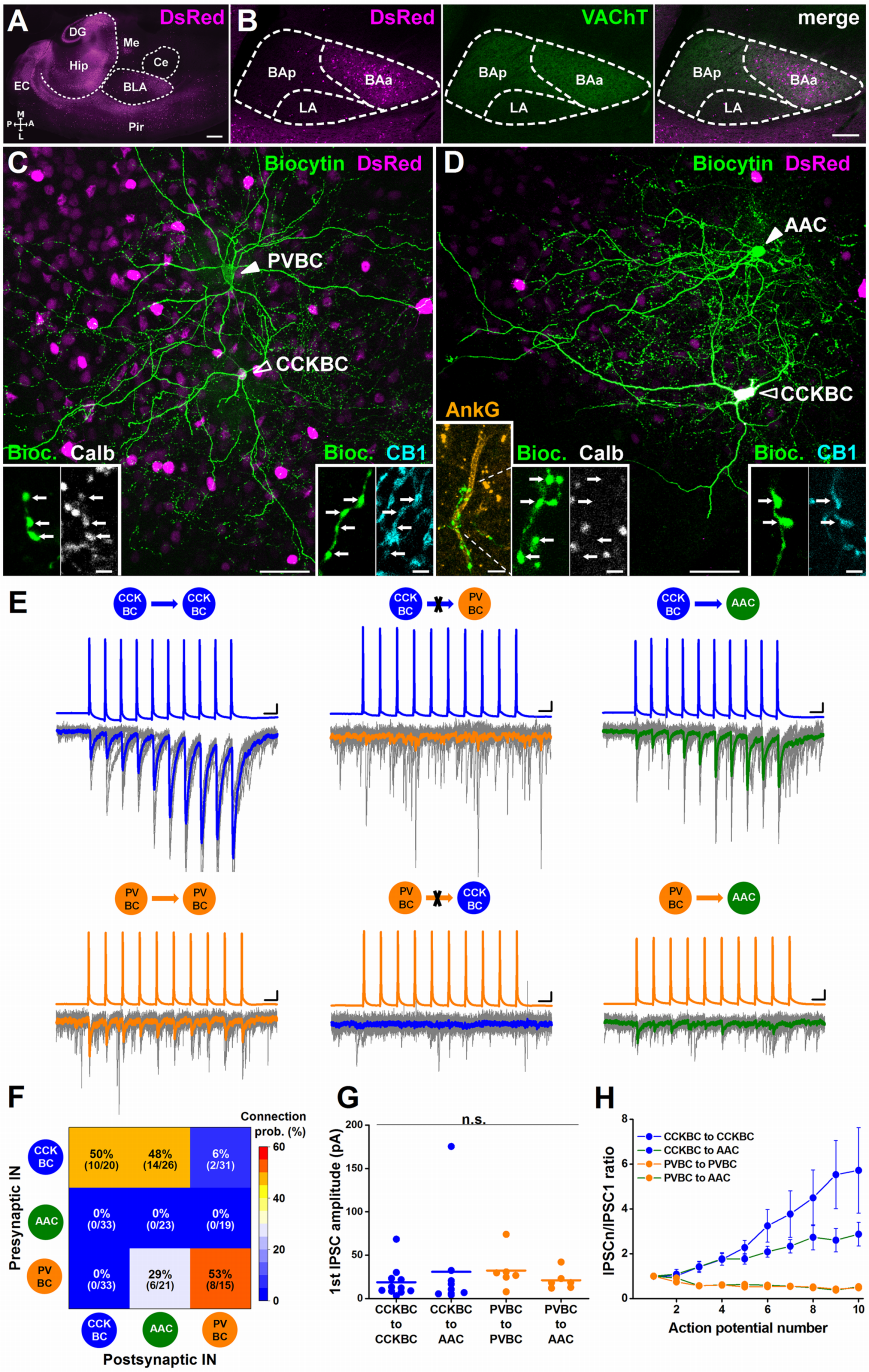
Cholecystokinin-expressing basket cells (CCKBCs) and parvalbumin-containing basket cells (PVBCs) form 2 parallel inhibitory circuits, but both innervate axo-axonic cells (AACs). (A) Horizontal section taken from the amygdala region of a transgenic mouse expressing red fluorescent protein under the control of the Cck promoter (CCK-DsRed). BLA, basolateral amygdala complex; EC, entorhinal cortex; Hip, hippocampus; DG, dentate gyrus; Me, medial amygdala; Ce, central amygdala; Pir, piriform cortex. Scale: 500 μm. (B) Red fluorescent protein (DsRed) expression and cholinergic fibers visualized by immunostaining against the vesicular acetylcholine transporter (VAChT) parcel out the basolateral amygdala complex into 3 parts: the anterior part of the basal amygdala (BAa) contains a pronounced DsRed signal and strong VAChT immunoreactivity, the posterior part of the BA (BAp) is characterized by low and high levels of DsRed and VAChT expression, respectively, and the lateral amygdala (LA) has low levels of both signals. Scale: 200 μm. (C) Maximum z intensity projection image of a PVBC and a CCKBC recorded in the BAa of a PV-eGFP x CCK-DsRed double transgenic mouse. The soma of the PVBC showed eGFP, but not DsRed signal (solid arrowhead), and its boutons were immunostained for calbindin (Calb, bottom left panels) that typifies PVBCs. The axon terminals of the CCKBC, which soma contained DsRed but not eGFP signal (open arrowhead), were immunoreactive for cannabinoid receptor type 1 (CB1) (bottom right panels). (D) Maximum z intensity projection image of an AAC and a CCKBC recorded simultaneously in the BAa. The axon collaterals of the AAC, which soma contained eGFP but not DsRed signal (solid arrowhead), formed close apposition with the axon initial segments visualized by immunostaining against ankyrin G (AnkG) and lacked Calb (bottom left panels), typical for AACs. The boutons of the CCKBC (open arrowhead), which soma showed DsRed but not eGFP signal, expressed CB1 (bottom right panels), characteristic for CCKBCs. Scale: 50 μm, insets 2 μm. (E) Representative traces from whole-cell paired recordings from post hoc identified different interneuron→interneuron pairs. Ten superimposed consecutive traces are in gray, averages are in bold. Scale: y: 10 mV/10 pA, x: 20 ms. (F) Connection probability matrix of interneuron→interneuron synaptic coupling was obtained by paired recordings. (G) Comparison of the amplitude of inhibitory postsynaptic currents (IPSCs) (18.8 ± 5.5 pA, *n* = 11 CCKBC→CCKBC pairs; 30.8 ± 18.3 pA, *n* = 9 CCKBC→AAC pairs; 32.2 ± 9.0 pA, *n* = 6 PVBC→PVBC pairs; 21.1 ± 4.5 pA, *n* = 6 PVBC→AAC pairs, Kruskal-Wallis ANOVA *p* = 0.2) (S1 Data). Each data point on the plot is an average obtained from a pair recording, and lines represent mean. (H) Comparison of the short-term plasticity of the different types of interneuron→interneuron connections (IPSC_10_/IPSC_1_: 5.72 ± 1.91, *n* = 7 CCKBC→CCKBC pairs; 2.87 ± 0.52, *n* = 7 CCKBC→AAC pairs; 0.48 ± 0.05, n = 6 PVBC→PVBC pairs; 0.53 ± 0.08, *n* = 4 PVBC→AAC pairs. Kruskal-Wallis ANOVA, *p* < 0.001) (S1 Data). Data are presented as mean ± SEM.

We focused our investigations on the BA, predominantly on its anterior part (Fig 1A and 1B). To verify that, in the BA, CCKBCs and PVBCs innervate their own kind as in other cortical regions [20–24], we performed paired whole-cell recordings from 2 interneurons in slices prepared from mice expressing red fluorescent protein under the control of the Cck promoter (CCK-DsRed) and from mice which expressed enhanced green fluorescent protein under the control of the Pvalb promoter (PV-eGFP), respectively [37]. In agreement with previous data, a monosynaptic connection from CCKBCs to CCKBCs and from PVBCs to PVBCs could be detected with high probability with a protocol evoking a train of action potentials in the presynaptic interneuron and monitoring the unitary events in the postsynaptic interneuron (Fig 1E and 1F). These observations indicate that both BC types form heavily interconnected networks within their own population. In amygdalar slices containing enhanced green fluorescent protein (eGFP)-expressing cells, we also identified a unidirectional connectivity from PVBCs onto AACs (Fig 1E and 1F), in line with previous observations obtained in the hippocampus [21]. The 2 PV-expressing interneuron types were distinguished by their calbindin content, because this Ca^2+^ binding protein is typically present in PVBCs but not in AACs in the BA (Fig 1C and 1D; [34]). In many cases, the identity of AACs was further strengthened by investigating the close appositions of biocytin-labeled varicosities along the axon initial segments visualized by immunostaining against ankyrin G. This latter method allows unequivocal identification of AACs in cortical structures [34, 38]. To study the synaptic connectivity between CCKBCs and PVBCs, we crossed the CCK-DsRed and PV-eGFP mice and simultaneous recordings were obtained in slices prepared from the double transgenic mice (S1A Fig). No monosynapic connection could be detected from PVBCs onto CCKBCs (0 out of 33 trials, Fig 1C, 1E and 1F), while we observed in 2 cases out of 31 trials that CCKBCs gave rise to synaptic input onto PVBCs with a small peak amplitude on average (12.8 pA and 30.6 pA)(Fig 1C, 1E and 1F). In contrast, unidirectional connectivity from CCKBCs onto AACs was observed with a high likelihood (14 out of 26 trials; Fig 1D–1F). These in vitro results indicate that CCKBCs and PVBCs typically do not innervate each other but form 2 functionally independent, parallel GABAergic circuits (Fig 1E). This active avoidance of cross-connectivity between the 2 BC types is strengthened by the fact that they both innervated the intermingled population of AACs expressing PV (Fig 1E and 1F).

Interestingly, the amplitude of unitary inhibitory postsynaptic currents (uIPSCs) in response to the first action potentials in each train was similar irrespective of the nature of the pre- or postsynaptic interneurons (Fig 1G, S1 Data). A marked difference, however, was seen in the short-term dynamics of synaptic transmission. The output synapses of CCKBCs and PVBCs showed strong facilitation and modest depression, respectively (Fig 1H, S1 Data). In addition, we found some differences in other characteristics of synaptic transmission, including the latency and rise times (S1 Fig, S9 Data). In summary, out of 9 possible cases, 4 monosynaptic connections were found to be realized functionally among the 3 types of interneurons, suggesting a highly specific connectivity matrix among GABAergic cells targeting the perisomatic region of PNs (Fig 1F).

In other cortical regions, the presence of electrical coupling between interneurons of the same type is characteristic [39, 40], possibly promoting synchronous activities [41, 42]. To test whether gap junction coupling exists between perisomatic inhibitory cells in the BA, we injected hyperpolarizing current steps into an interneuron, and responses were monitored in the other one. We found that interneurons of the same type were often coupled electrically (S2 Fig, S10 Data). Notably, a high probability of gap junction coupling was observed between AACs that are not coupled via synaptic junctions (Fig 1E, S2 Fig, S10 Data). These data strengthen the above observation that CCKBCs and PVBCs form 2 parallel circuits in which these GABAergic cells are massively interconnected via both synapses and gap junctions within their own type.

**Fig 2:**
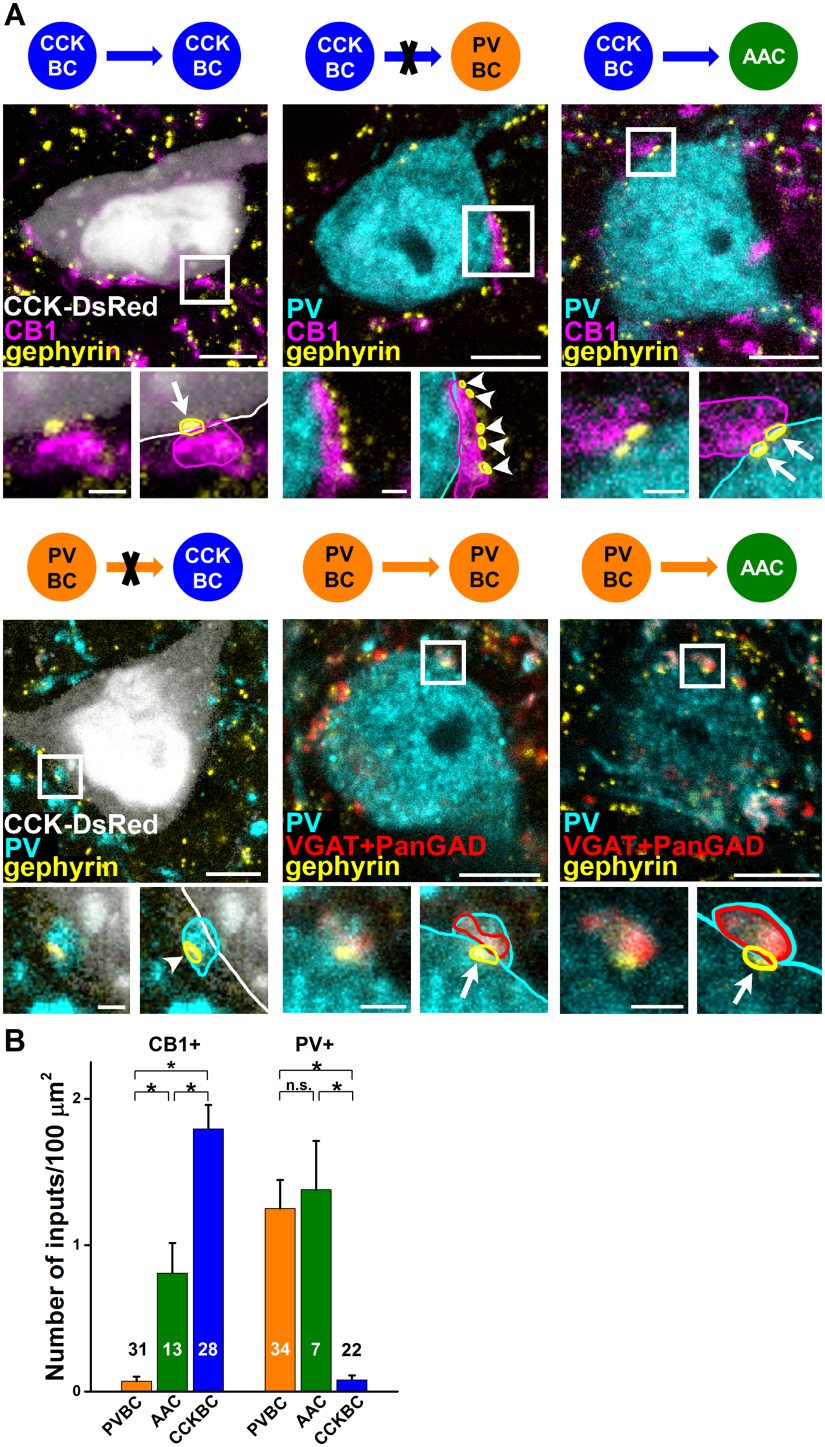
Cholecystokinin-expressing basket cells (CCKBCs) and parvalbumin-containing basket cells (PVBCs) avoid innervating each other but establish contacts on axo-axonic cells (AACs). (A) Quantification of somatic cannabinoid receptor type 1 (CB1)- and parvalbumin (PV)-expressing inhibitory inputs (arrows) onto CCKBCs, PVBCs, and AACs using fluorescent immunostainings against gephyrin, CB1, PV, and vesicular gamma-aminobutyric acid (GABA) transporter (VGAT)/glutamate decarboxylase 65/67 (PanGAD). Axon terminals contacting the interneuron soma and other structures are labeled with arrows and arrowheads, respectively. Delineation of the cell bodies, terminals, and gephyrin puncta are shown for clarity. Scales: 5 μm and 1 μm. (B) Density of CB1- and PV-immunostained terminals contacting the different types of perisomatic region-targeting interneurons (CB1 on PVBC: 0.06 ± 0.03; CB1 on AAC: 0.81 ± 0.21; CB1 on CCKBC: 1.79 ± 0.16; Kruskal-Wallis ANOVA, *p* < 0.001; PVBC versus AAC *p* < 0.001, PVBC versus CCKBC *p* < 0.001, AAC versus CCKBC *p* = 0.002, Mann–Whitney *U* test; PV on PVBC: 1.25 ± 0.19; PV on AAC: 1.38 ± 0.33; PV on CCKBC: 0.08 ± 0.03, Kruskal-Wallis ANOVA, *p* < 0.001, PVBC versus AAC *p* = 0.36, PVBC versus CCKBC *p* < 0.001, AAC versus CCKBC *p* < 0.001, Mann–Whitney *U* test)(S2 Data), labels on columns show the number of examined somata in each group. Mean ± SEM.

To confirm these in vitro electrophysiological data with an independent method, we investigated the anatomical substrate for the connectivity among interneurons in vivo using immunocytochemistry. The axon terminals of CCKBCs were visualized with an antibody developed against CB1 [34, 43], while PV expression detected by immunostaining was used to label boutons of PVBCs [34, 44]. We noticed that CB1-expressing boutons often contacted the somata of CCKBCs, while PV-containing axonal boutons, separated from other PV-expressing profiles by their co-expression of vesicular GABA transporter (VGAT) and glutamate decarboxylase 65/67 (PanGAD), often apposed PV-immunolabeled somata. In both cases, gephyrin, an anchoring protein of GABA_A_ receptors [45], was present at the soma facing the boutons, implying that both PVBCs and CCKBCs formed synaptic contacts with other interneurons of their own kind (Fig 2A, S3 Fig). In sharp contrast, we found that CB1-containing terminals almost completely avoided PVBC somata, and vice versa; CCKBC cell bodies were only rarely contacted by PV-immunolabeled terminals. In some cases, we could notice CB1- and PV-expressing varicosities in close vicinity to PV- and CCK-containing interneuron somata, respectively. However, gephyrin puncta were characteristically not present between these immunostained profiles but were instead found on the opposite side of the boutons, implying that the connections were established on unlabeled neighboring structures (Fig 2A, S3 Fig). These data support our in vitro results showing that the 2 BC types do not prefer to innervate each other. In contrast, and in line with our electrophysiological observations, the somata of AACs, separated from PVBCs based on their calbindin content (S3 Fig, [34]), received synaptic inputs from both CB1- and PV-containing boutons, as indicated by the presence of gephyrin labeling at the closely apposed boutons expressing CB1 or PV (Fig 2A, S3 Fig). Quantification showed that CCKBCs and PVBCs only occasionally innervate each other, but they targeted other BCs of the same type as well as AACs (Fig 2B, S2 Data).

Collectively, these results clearly show that CCKBCs and PVBCs form 2 functionally parallel GABAergic circuits in the BA.

### CCKBCs and PVBCs can both potently inhibit PN spiking in the BA

**Fig 3:**
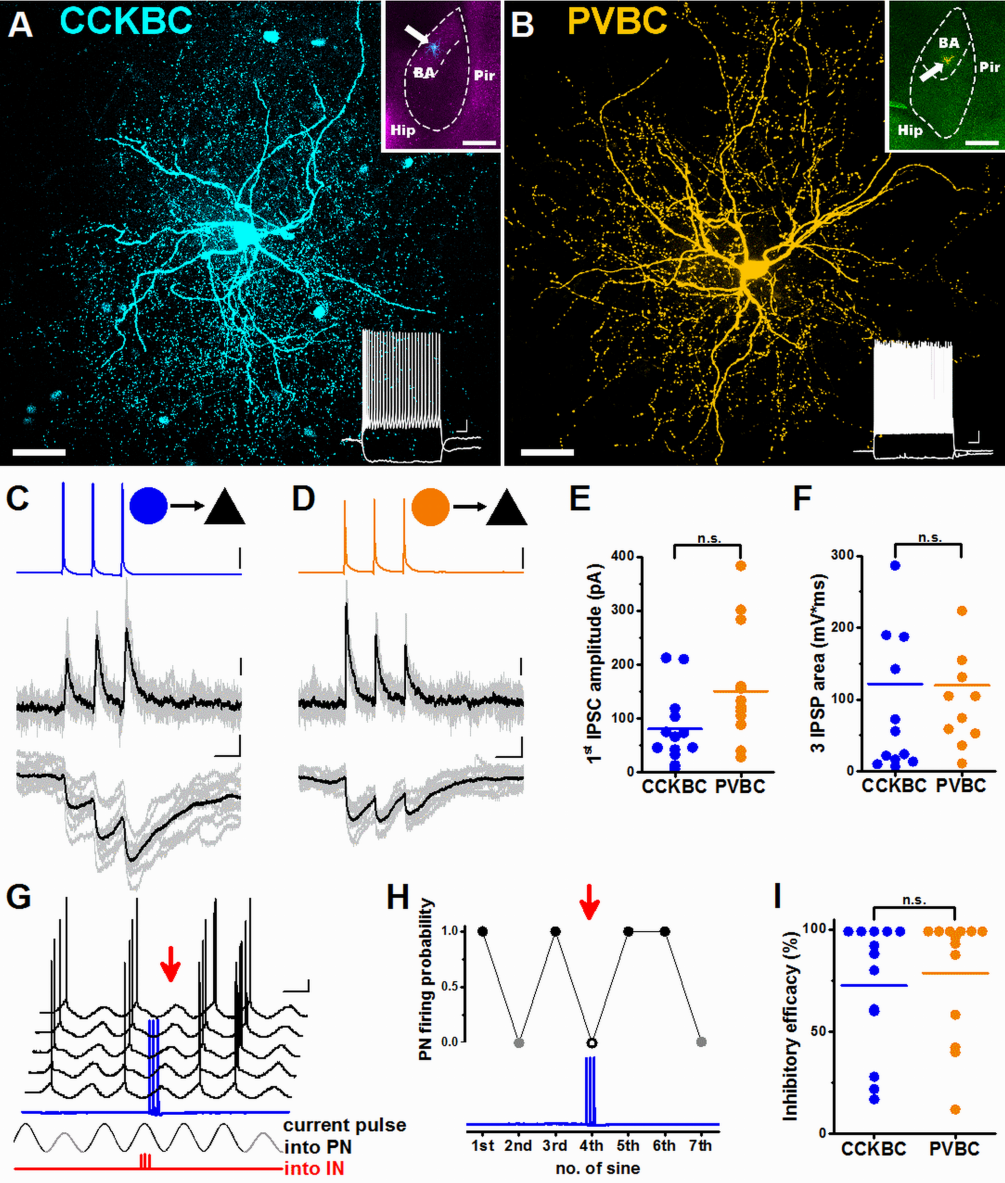
Similar potent inhibition of principal neuron (PN) activity by cholecystokinin-expressing basket cells (CCKBCs) and parvalbumin-containing basket cells (PVBCs). Maximum z intensity projection image of a CCKBC (A) and a PVBC (B). Scales: 50 μm. Upper insets in (A) and (B) indicate the positions of the recorded basket cells (BCs, white arrows) in horizontal slices prepared from mice expressing red fluorescent protein under the control of the Cck promoter (CCK-DsRed) and mice expressing enhanced green fluorescent protein under the control of the Pvalb promoter (PV-EGFP), respectively. Hip, hippocampus; BA, basal amygdala; Pir, piriform cortex. Scale for the insets: 500 μm. Lower insets show the firing patterns of the same BCs in response to a step current injection (+400 and −100 pA). Scales: 10 mV, 100 ms. (C, D) Representative inhibitory postsynaptic current (IPSC, middle traces) and potential (IPSP, lower traces) recordings in a CCKBC (blue)→PN (black) pair (C) or in a PVBC (orange)→PN pair (D) in response to 3 action potentials at 30 Hz (upper traces). Ten superimposed consecutive traces are in gray, average in black. Scales for (C) and (D): y: 20 mV/20 pA/0.5 mV, x: 20 ms. (E) CCKBCs and PVBCs give rise to IPSCs with similar amplitude (80.53 ± 18.36 pA, *n* = 13 CCKBC pairs and 150.38 ± 30.24 pA, *n* = 13 PVBC pairs, Mann–Whitney *U* test, *p* = 0.073) (S3 Data), and (F) 3 IPSPs with the similar area (121.83 ± 43.71 mV*ms, *n* = 13 CCKBC pairs and 119.78 ± 30.35 mV*ms, *n* = 11 PVBC pairs, Mann–Whitney *U* test, *p* = 0.45) (S3 Data) recorded in PNs. (G) Sinusoidal current trains with peak-to-peak amplitude of 30 pA (gray cycles) and 50 pA (black cycles) were injected into a PN to initiate firing, and 3 action potentials were evoked in the presynaptic CCKBC (blue) at the fourth cycle (red arrow). Schematic representation of the injection of 3 current pulses into the presynaptic interneuron (IN) at 30 Hz is shown in red. Voltage traces are offset for clarity. Scale: 10 mV, 200 ms. (H) Raw data of the experiments are shown in (G). Black and gray dots refer to the firing probability observed at the sinusoid current amplitudes of 50 pA and 30 pA, respectively. An open circle indicates the cycle when the presynaptic interneuron fired 3 action potentials. (I) Comparison of the inhibitory efficacy of CCKBCs and PVBCs (72.53 ± 8.80%, *n* = 13 CCKBC pairs and 78.70 ± 8.29%, *n* = 13 PVBC pairs, Mann–Whitney *U* test, *p* = 0.63) (S3 Data). Each data point on the plots represents an average obtained in a pair recording, and lines represent means.

Our data raises an intriguing question of whether the BCs belonging to the 2 distinct GABAergic networks provide PNs with similar or distinct inhibitory input. Therefore, we examined the characteristics of the output synapses of PVBCs and CCKBCs and their effects on the spiking of postsynaptic PNs, using paired recordings in vitro. Three action potentials evoked at 30 Hz (a physiologically relevant spiking activity [33]) in a presynaptic BC resulted in postsynaptic responses in a PN (Fig 3A–3D). There was a high and similar probability to find a monosynaptically connected pair in the case of both BC types (CCKBC→PN, 81.25%, *n* = 16 tested pairs; PVBC→PN, 92.86%, *n* = 14 tested pairs; Fisher’s exact test, *p* = 0.34). In addition, we found that many properties of unitary inhibitory postsynaptic currents (IPSCs) and potentials (IPSPs) evoked by the first action potentials of the trains were also similar (Fig 3E and 3F, S3 Data, S4 Fig, S11 Data). As in the hippocampus [12, 14], 3 action potentials evoked in PVBCs resulted in depressing responses in the PNs, while there was no obvious change in the amplitude of IPSC/IPSPs evoked by spike trains in CCKBC→PN pairs at the population level (Fig 3C and 3D). However, this difference in short-term plasticity was not apparent in the IPSP summation, as the area under IPSPs evoked by 3 spikes in the 2 BC types were indistinguishable (Fig 3F, S3 Data). The similarities in the unitary events originating from the 2 BC types implied that these GABAergic cells may have comparable effects on the spiking of PNs.

To test this prediction, we injected sinusoidal current trains into the PNs near their firing threshold, and 3 spikes at 30 Hz were evoked in the presynaptic BCs at the peak of a sinusoidal wave, the point where PNs spiked with the highest probability (Fig 3G). This reproducible approach allowed us to calculate the efficacy of inhibition for both GABAergic cell types by comparing the firing probability of PNs during evoked BC activity and during control epochs, during which the presynaptic BC did not fire (Fig 3G and 3H). This analysis fully supported our expectation based on the unitary event properties, namely, that the 2 BC types inhibited PN firing with equal efficacy (Fig 3I, S3 Data), similarly to that observed at a young age (S5 Fig, S12 Data [37]).

These results show that in this amygdala network, PVBCs and CCKBCs provide similarly effective synaptic inhibition onto their neighboring PNs.

### CCKBCs and PVBCs receive excitatory inputs with distinct properties

**Fig 4:**
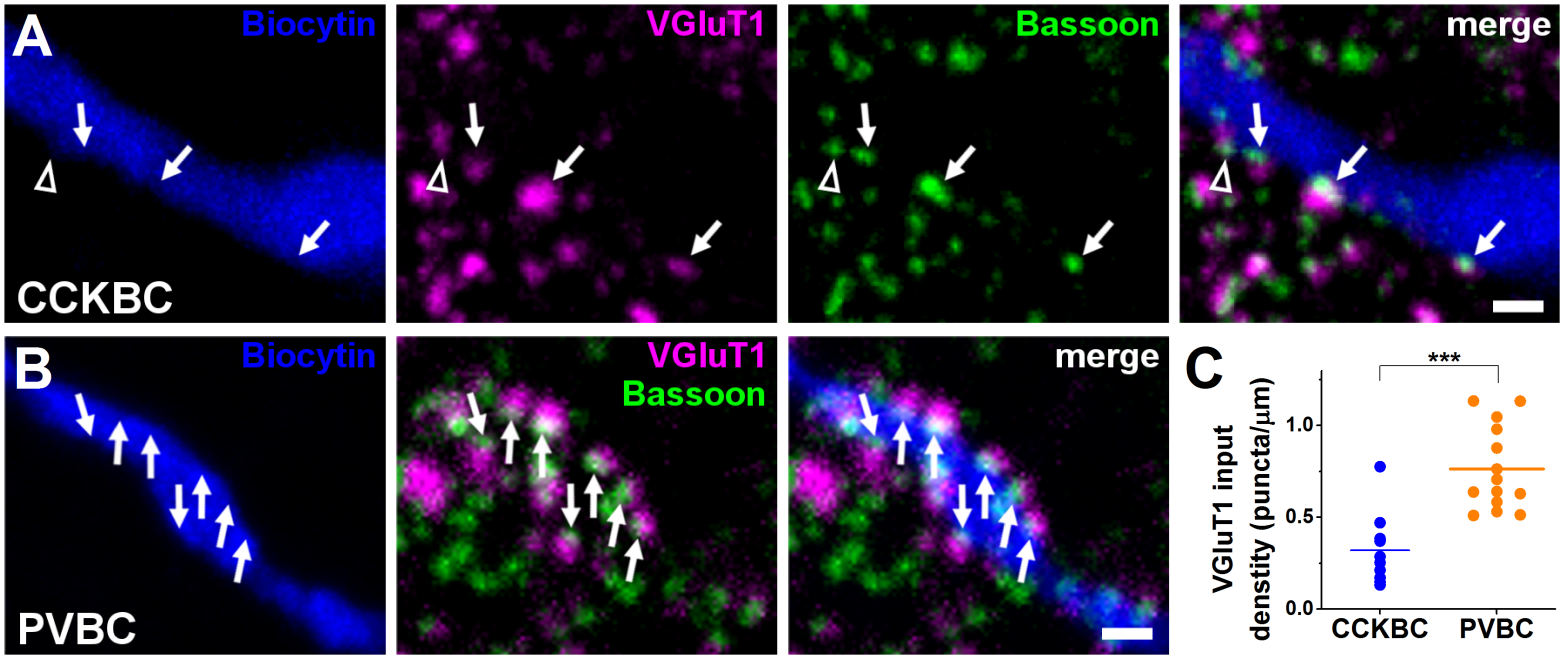
Parvalbumin-containing basket cells (PVBCs) receive a higher number of excitatory inputs than cholecystokinin-expressing basket cells (CCKBCs). VGluT1-immunopositive puncta opposing the dendrite of a CCKBC (A) and a PVBC (B) and containing bassoon staining were regarded as a contact (arrows). Open arrowhead shows a vesicular glutamate transporter 1 (VGluT1)-immunoreactive bouton in which bassoon didn't oppose the biocytin-labeled dendrite, presumably contacting a neighboring structure. Scale: 1 μm. (C) Each data point on the plot represents an average density obtained on a dendritic segment, lines represent mean. Biocytin-labeled dendritic segments of 5 CCKBCs and 6 PVBCs were investigated. (CCKBC: 0.32 ± 0.06, PVBC: 0.76 ± 0.06, Mann–Whitney *U* test, ***p < 0.001) (S4 Data).

If the 2 parallel BC networks have similar potency to alter the activity of PN populations by controlling their spiking, then, to function largely independently, CCKBCs and PVBCs must receive distinct excitatory inputs, a difference that can be reflected both in the number of synapses and their magnitude. To reveal whether the 2 BC types receive a different number of excitatory inputs, we first estimated the density of glutamatergic axonal varicosities forming bassoon-apposing contacts with the intracellularly-labeled BCs. Here, bassoon, a protein in the active zone of synapses [46], was used to visualize the presence of functional synapses at the light microscopic level. This analysis showed that the dendrites of PVBCs were covered more than 2 times more densely by vesicular glutamate transporter 1 (VGluT1)-expressing boutons than the dendrites of CCKBCs (Fig 4, S4 Data).

**Fig 5:**
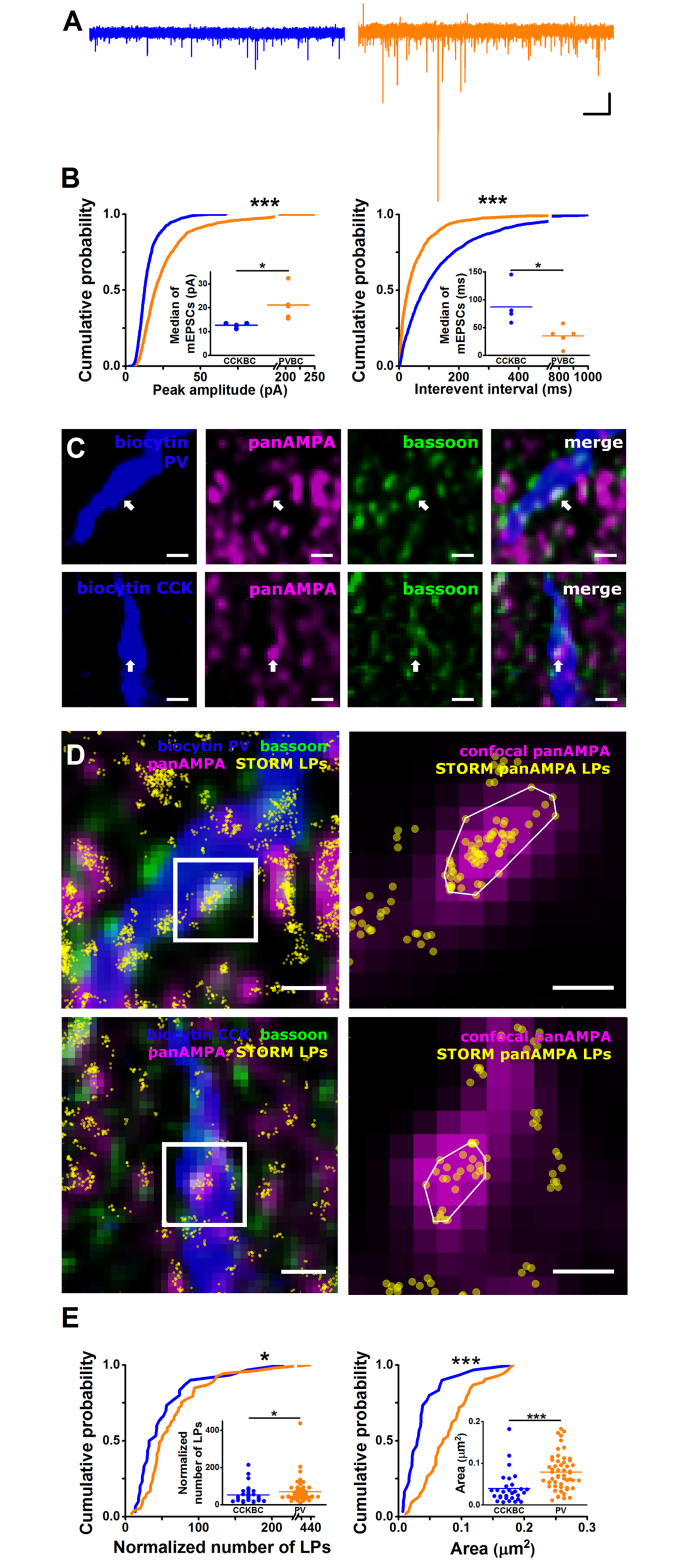
Excitatory inputs of the 2 basket cell (BC) types have distinct properties. (A) Representative traces depicting miniature excitatory postsynaptic currents (mEPSCs) in the presence of 0.5 μM tetrodotoxin (TTX) in a cholecystokinin-expressing basket cells (CCKBC) (left, blue) and in a parvalbumin-containing basket cells (PVBC) (right, orange) (holding potential, –65 mV). Scales: x: 0.5 s, y: 30 pA. (B) Cumulative probability distributions of mEPSC peak amplitudes (left) and interevent intervals (right) obtained in CCKBCs (blue, *n* = 5) and PVBCs (orange, *n* = 5). PVBCs receive mEPSCs with larger amplitude and at higher frequency than CCKBCs (Kolmogorov-Smirnov test, ***p < 0.001). Data points in the insets represent the median of the cumulative distributions obtained for each cell, and lines indicate means (peak amplitude: CCKBCs, 12.62 ± 0.43 pA, *n* = 5, PVBCs, 21.19 ± 3.01 pA, *n* = 5; interevent interval: CCKBCs, 86.97 ± 15.03 ms, *n* = 5; PVBCs, 34.87 ± 8.02 ms, *n* = 5; Mann–Whitney *U* test, *p < 0.05) (S5 Data). (C) Examples of deconvolved confocal images showing bassoon-apposing puncta immunostained for α-amino-3-hydroxy-5-methyl-4-isoxazolepropionic acid (AMPA) receptors in a dendritic portion of a PVBC (arrows, upper panels) and a CCKBC (arrows, lower panels). Scale bars: 0.4 μm. (D) Left, the same dendritic fragments shown in (C) with the superimposed stochastic optical reconstruction microscopy (STORM) images obtained for AMPA receptor staining (yellow), aligned and filtered using VividSTORM. Right, higher magnification of representative puncta depicting AMPA receptors along the interneuron dendrites, showing the deconvolved confocal image aligned with the STORM image. Each yellow sphere corresponds to a localization point (LP), while the white lines delineate the 2D convex hull area based on the AMPA LP clusters. Scales: left 0.5 μm, right 0.2 μm. (E) Cumulative probability distributions of the normalized number of LPs (left) and 2D convex hull area (right) obtained in CCKBCs (*n* = 30 puncta, blue) and PV-expressing interneurons (*n* = 53 puncta, orange). AMPA receptor clusters on CCKBC dendrites are smaller and contain lower number of LPs than those observed on parvalbumin (PV)-immunoreactive dendrites (normalized number of LPs: CCKBCs, 53.13 ± 8.58, PV-containing interneurons 69.28 ± 8.90; 2D convex hull area: CCKBCs, 0.04 ± 0.007 μm^2^, PV-containing interneurons, 0.08 ± 0.006 μm^2^; Kolmogorov-Smirnov test, *p < 0.05, ***p < 0.001) (S5 Data). Each data point in insets represents a cluster, and lines indicate means (Mann–Whitney *U* test, *p < 0.05, ***p < 0.001).

To strengthen these findings that the 2 BC types receive different number of excitatory inputs, we recorded miniature (i.e., quantal) excitatory postsynaptic currents (mEPSCs) in the presence of 0.5 μM tetrodotoxin and investigated the properties of single events. By analyzing the interevent interval distributions of mEPSCs, we observed a marked difference (Fig 5A and 5B, S5 Data). In CCKBCs, the time intervals between mEPSCs (i.e., the reciprocal of the frequency) were significantly longer than between those events recorded in PVBCs. This data along with the anatomical results (Fig 4, S4 Data) confirms that significantly more glutamatergic inputs are received by PVBCs than by CCKBCs.

To assess the potential differences in the magnitude of quantal excitatory inputs of the 2 BCs, we examined the amplitudes of mEPSCs. We found that the mEPSC amplitudes recorded in CCKBCs were substantially smaller than those obtained in PVBCs, resulting in a significant difference in the amplitude distributions of mEPSCs (Fig 5A and 5B, S5 Data). Importantly, mEPSCs in CCKBCs not only had smaller amplitudes but also showed a low variance across recordings (mini recordings: 12.62 ± 0.43 pA, *n* = 5 cells, Coefficient of Variation, [CV] = 0.07), indicating that the number of receptors between release sites should vary in a narrow range. In contrast, mEPSCs in PVBCs had overall larger amplitudes and showed a more skewed distribution (mini recordings: 21.19 ± 3.01 pA, *n* = 5 cells, CV = 0.31, Fig 5B, S5 Data), showing that a large difference in the number of receptors among individual release sites should be typical for these BCs.

To support the conclusions of our mEPSC amplitude analysis, we estimated the α-amino-3-hydroxy-5-methyl-4-isoxazolepropionic acid (AMPA) receptor content at individual synapses along the CCK- and PV-expressing interneuron dendrites using super-resolution microscopy (3D-STORM, Fig 5C–5E, S5 Data). Although, at present, it is unclear how many localization points (LPs) represent a protein, preventing us from quantifying the precise number of receptors in a given synapse, the comparison of the normalized numbers in LPs between 2 samples still provides valuable information of the relative receptor content in both populations. These investigations uncovered that the normalized number of LPs, representing AMPA receptors at individual clusters along the CCKBC dendrites, was significantly lower than those observed along the interneuron dendrites expressing PV (Fig 5E, S5 Data). In addition, there was a significant difference in the area of LP clusters along the dendrites of 2 interneuron types (Fig 5E, S5 Data).

These anatomical and electrophysiological results collectively demonstrate that overall, CCKBCs and PVBCs receive both quantitatively and qualitatively distinct excitatory inputs.

### BA PNs distinctly innervate the 2 BC types

**Fig 6:**
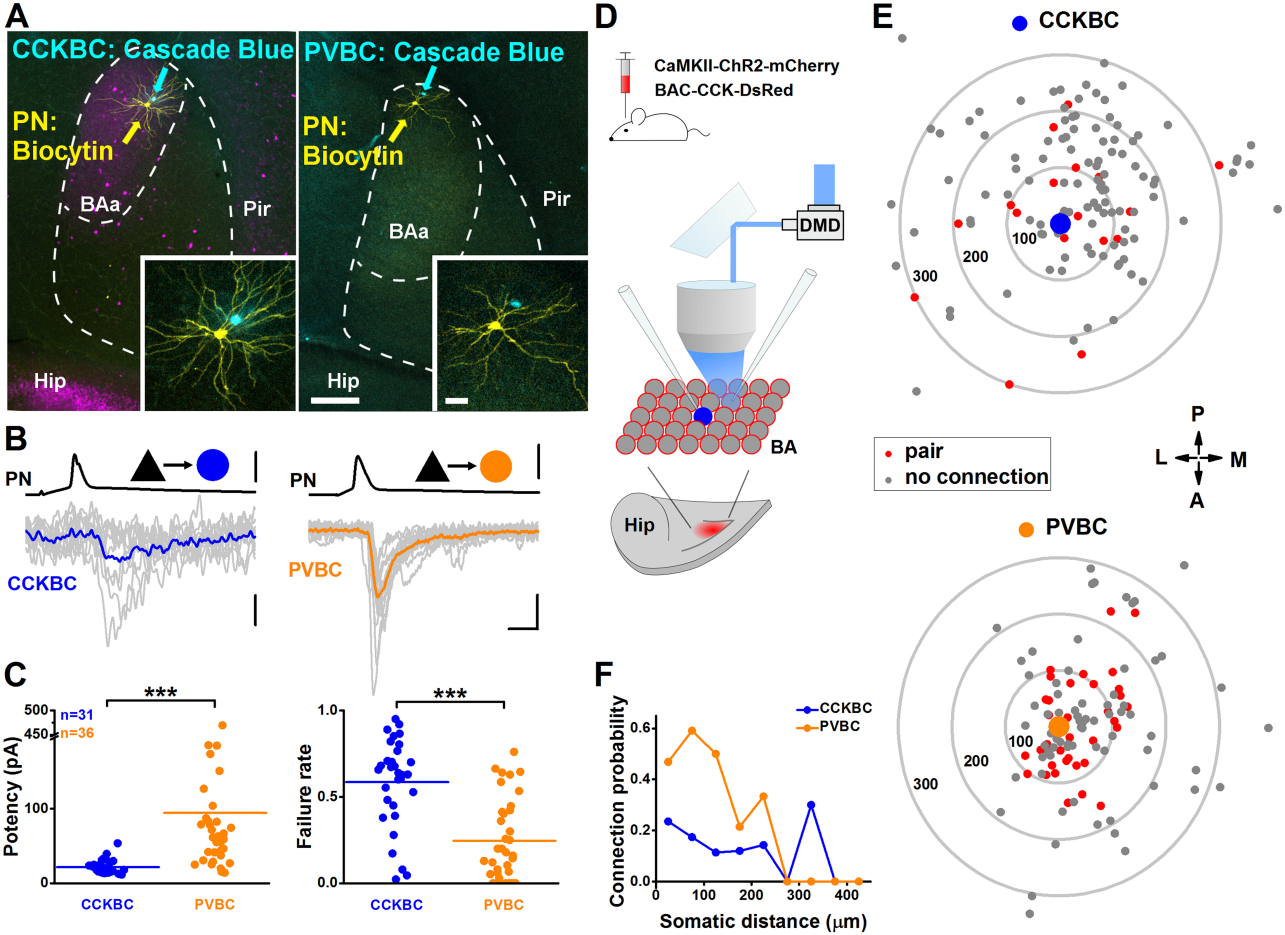
Principle neurons (PNs) give rise to distinct excitatory synaptic inputs onto the 2 basket cell (BC) types. (A) Panoramic images showing a cholecystokinin-expressing basket cell (CCKBC)→PN (left) and a parvalbumin-containing basket cell (PVBC)→PN (right) pair in the anterior part of the basal amygdala (BA). Interneurons were filled with Cascade Blue and PNs with biocytin. Insets show a higher magnification of the cell pairs recorded. Scales: 250 μm. (B) Representative traces of unitary excitatory postsynaptic currents (EPSCs) evoked by action potentials in a PN→CCKBC (upper traces) and a PN→PVBC pair (lower traces). Ten superimposed consecutive traces are in gray, and averages are in blue and orange. Scales: x: 2 ms; PN→CCKBC, y: 80 mV/8 pA; PN→PVBC, y: 90 mV/30 pA. (C) PVBCs receive unitary excitatory postsynaptic currents (uEPSCs) from local PNs with larger amplitude and lower failure rate than CCKBCs (potency, CCKBC: 21.5 ± 1.6 pA, PVBC: 94.25 ± 15.08 pA; failure rate, CCKBC: 0.58 ± 0.04, PVBC: 0.25 ± 0.04; Mann–Whitney *U* test, ***p < 0.001) (S6 Data). Each data point on the plots represents an average obtained in a pair recording, and lines represent means. (D) Schematic illustration of the experimental design to excite PNs using blue light. mCherry-containing construct was injected into the BA of transgenic mice (bottom drawing illustrates a horizontal slice showing the expression site in red). Among adeno-associated virus (AAV)-infected PNs (red circles), a CCKBC (blue) was recorded in whole-cell mode, while its excitatory input from neighboring PNs was tested by light illumination and/or whole-cell recording. BA, basal amygdala; Hip, hippocampus. (E) Connectivity map. Concentric circles indicate Δ100 μm distance from the interneuron in the center (S6 Data). Each dot represents a tested PN soma location. (F) PVBCs receive excitatory synaptic inputs from their neighboring PNs with high probability, while CCKBCs are innervated by PNs via their local collaterals with low probability, independently of the inter-somatic distance (S6 Data).

To directly test whether a defined excitatory input can indeed distinctly target CCKBCs and PVBCs, we have chosen to study the synaptic properties of local afferents of BA PNs, because it is known that PNs in cortical regions readily innervate interneurons in their vicinity [47, 48], which is a key determinant of microcircuit operation [15, 49]. As a first step, we investigated the properties of unitary events from individual PNs onto BCs. We made in vitro paired recordings when current pulses were injected into the presynaptic PN to evoke action potentials, and the postsynaptic responses were detected in the BC. We found that the average potency and failure rate of unitary excitatory postsynaptic currents (uEPSCs) were significantly larger and lower, respectively, in PVBCs than in CCKBCs (Fig 6B and 6C, S6 Data), as well as all other synaptic parameters tested (S6 Fig, S13 Data). In addition, single PNs expressing ChR2-mCherry were optogenetically excited and tested the connection between PNs and BCs in PV-eGFP or CCK-DsRed mice. In this latter case, action potentials monitored in loose-patch mode were evoked in the PNs by a spot of blue light illumination, while the postsynaptic responses in the BCs were recorded in whole-cell mode (S6A–S6D Fig). Since action potentials evoked by intracellular current injection and light stimulation resulted in uEPSCs with similar characteristics (S7E–S7J Fig, S14 Data), both methods were used to map the location of PNs that innervate BCs (S8 Fig, S15 Data). We found that excitatory cells were connected to PVBCs with a significantly higher likelihood than CCKBCs (S6A Fig, S13 Data). In addition, we noticed that the connection probability showed distinct distance-dependence (Fig 6E and 6F, S6 Data). The construction of a spatial map for PN→BC pairs uncovered that PVBCs were contacted preferentially by their neighboring PNs (<200–250 μm), but only rarely by more distal excitatory neurons, in line with data obtained in neocortical microcircuits [50, 51]. In contrast, PNs innervated CCKBCs with lower probability, but the chance to find a connected pair was distance-independent (Fig 6E and 6F, S6 Data).

**Fig 7:**
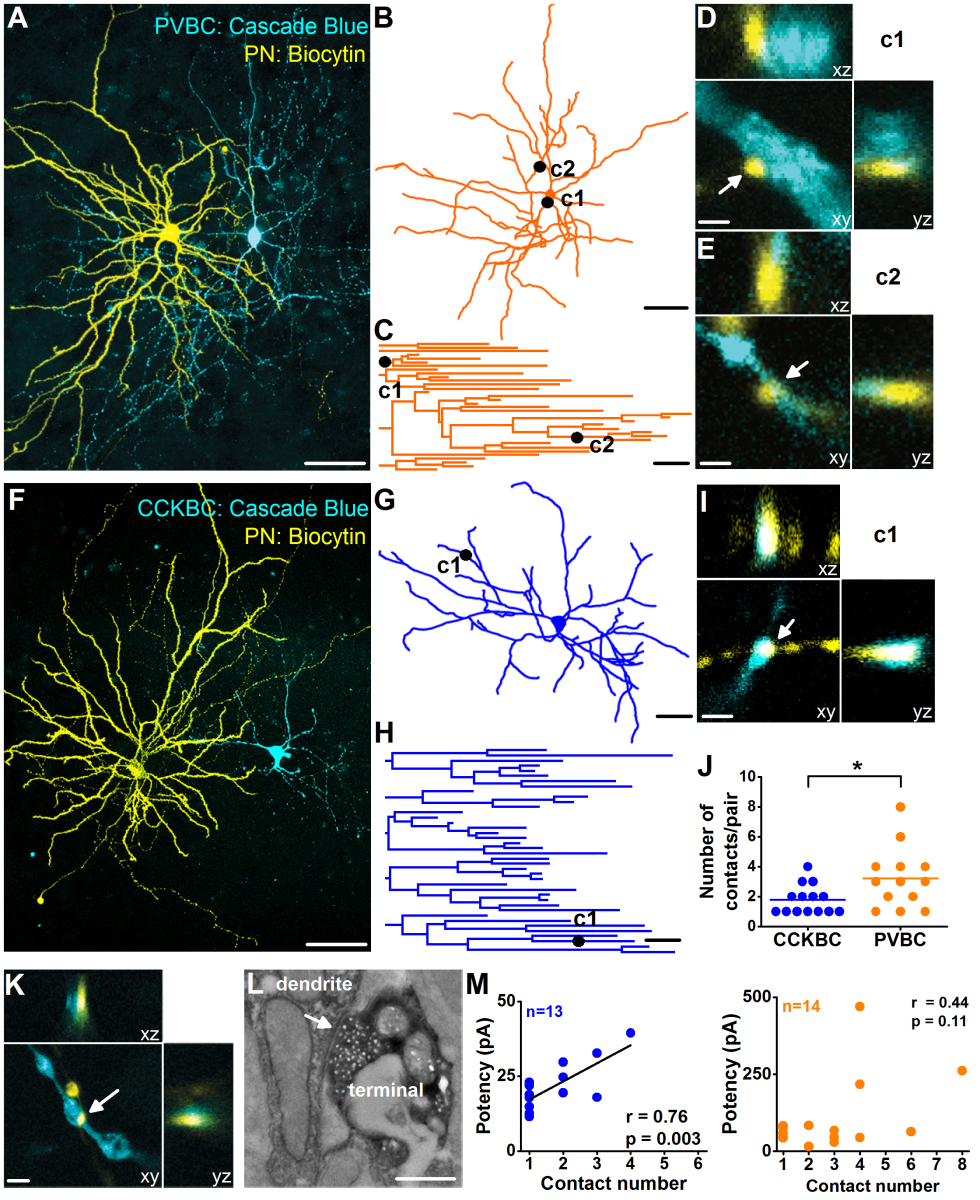
Principal neurons PNs innervate the 2 basket cell (BC) types differently. Maximum z intensity projection image of a representative PN→parvalbumin-containing basket cell (PVBC) pair (A) and a PN→cholecystokinin-expressing basket cells (CCKBC) pair (F). Cascade Blue was used to label the BCs, while biocytin was introduced into the PNs to visualize them with streptavidin-conjugated Alexa647. Scales for (A and F): 50 μm. Neurolucida reconstructions (B and G) and the resulted dendrogram analysis (C and H) of the PVBC and CCKBC shown in (A) and in (F), respectively, marking the location of connections 1 and 2 (c1 and c2) established by the monosynaptically connected presynaptic PNs. Scales for (B, C, G, H): 50 μm. (D and E) High-power magnification 3D confocal images of putative contacts c1 and c2, showing close appositions between the dendrite-targeting boutons of the PN (yellow) and the PVBC dendrites (blue). Scale: 2 μm. (I) High power magnification 3D confocal images of a putative contact c1, showing close appositions between the bouton of the PN (yellow) and the CCKBC dendrites (blue). Scales: 2 μm. (J) PNs establish significantly more contacts on PVBCs than on CCKBCs (1.79 ± 0.26, *n* = 14 PN→CCKBC pairs, 3.23 ± 0.57, *n* = 13 PN→PVBC pairs, Mann–Whitney *U* test, *p < 0.05) (S7 Data). Each data point on the plot was obtained from a pair recording, and lines represent means. (K) 3D confocal image showing a close apposition between a biocytin-labeled PN terminal (yellow) and a Cascade Blue-labeled BC dendrite (blue). Scale: 2 μm. (L) Electron microscopic analysis of the contact shown in (K) confirmed the presence of the synaptic junction (arrow). Scale: 500 nm. (M) A strong relationship was found between the number of contact sites and the unitary excitatory postsynaptic current (uEPSC) potency in the case of PN→CCKBC pairs, while no correlation could be observed in the case of PN→PVBC pairs (S7 Data).

Next, we performed an analysis of contact sites between intracellularly-labelled PNs and BCs using high-resolution confocal microscopy. This investigation revealed that, on average, twice as many contacts could be identified from single PNs onto PVBCs than onto CCKBCs (Fig 7A–7K, S7 Data). However, the location of the contact sites along the somato-dendritic membrane surface of both BCs was similar (PN→CCKBC pairs: 104.34 ± 12.67 μm, *n* = 25; PN→PVBC pairs: 89.28 ± 9.33 μm, *n* = 42; *p* = 0.28, Mann–Whitney *U* test). When the amplitude of unitary events was plotted as a function of the number of identified contact sites, a linear relationship was observed in the case of PN→CCKBC pairs, showing that an increase in the number of contacts results in a proportional increase in the unitary amplitude (Fig 7M, S7 Data). In contrast, no such relationship was found in the case of PN→PVBC pairs (Fig 7M, S7 Data). Moreover, in a distinct set of experiments, we compared the peak amplitudes of unitary events recorded in those pairs, in which only single contacts were detected. This analysis revealed a key difference in the local BC inputs at the level of individual synapses. CCKBCs received small (17.23 ± 4.45 pA [mean ± SD], *n* = 7) and surprisingly uniform (CV = 0.26) synaptic inputs from PNs, while uEPSCs conducted via single synapses in PVBCs were large and highly variable (78.09 ± 60.7 pA [mean ± SD], *n* = 9, CV = 0.78). These data are in line with the above results obtained by mEPSC amplitude and 3D-STORM analysis and support the conclusion that the inter-synaptic variance in the EPSC amplitude is much lower in CCKBCs than in PVBCs, which might indicate the distinct plastic properties of excitatory synapses on the 2 BC types.

Collectively, our findings show that BA PNs innervate CCKBCs and PVBCs via different principles, because PVBCs receive large and fast uEPSCs from their neighboring PNs that show a profound variance in their amplitude, while uEPSC amplitudes in CCKBCs are small and slightly variable unitary events that originate from PNs distributed more evenly in the BA. These results indicate that the 2 BC circuits in this cortical microcircuit can be distinctly operated by local excitatory drives.

### PVBCs are driven by lower PN activity levels than CCKBCs

**Fig 8:**
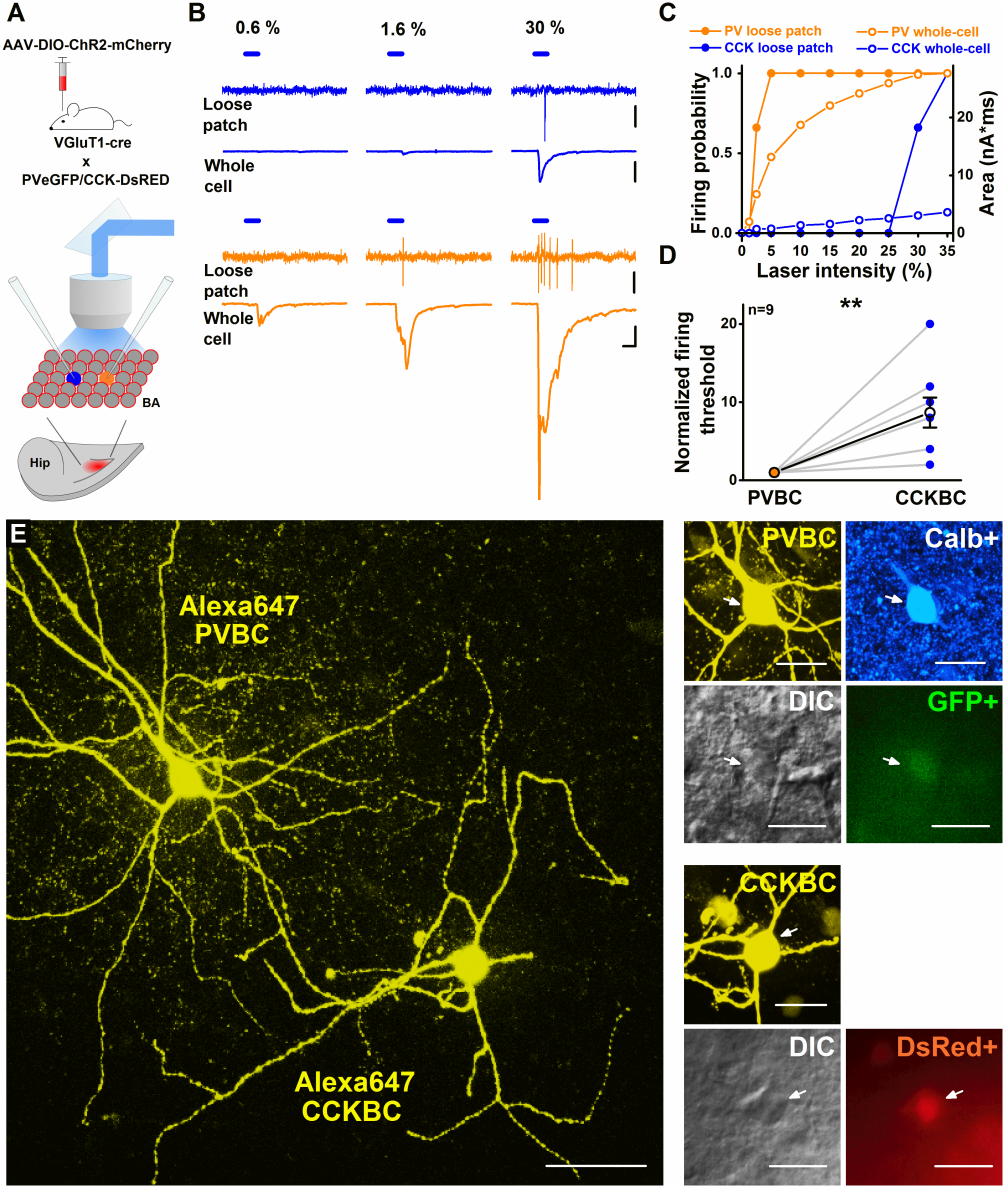
Spiking of parvalbumin-containing basket cells (PVBCs) requires a lower activity level of principal neuron (PN) population than cholecystokinin-expressing basket cells (CCKBCs). (A) Experimental design. mCherry-containing construct was injected into the basal amygdala (BA) of triple transgenic mice (bottom drawing illustrates a horizontal slice showing the expression site in red; Hip, hippocampus; BA, basal amygdala). Among adeno-associated virus (AAV)-infected PNs (red circles), a CCKBC (blue) and a PVBC (orange) were simultaneously recorded first in loose-patch mode, followed by recordings in whole-cell mode, while neighboring PNs were excited by light illumination. (B) Representative traces of loose-patch and whole-cell recordings show different spiking thresholds and, concomitantly, larger light-evoked responses in a PVBC (lower traces) compared to a CCKBC (upper traces) in response to PN stimulation at different light intensities. Averages calculated from 3 consecutive events are shown in blue and orange for the CCKBC and the PVBC, respectively. Scales: y: 25 pA, loose-patch; y: 200 pA whole-cell; x: 10 ms. (C) A summary graph of the experiment shown in (B); the firing probability (solid circles, left axis) and the integral of the light-evoked responses (open circles, right axis) detected in basket cells (BCs) upon gradually elevated stimulation intensities (S8 Data). (D) The activation threshold of PVBCs is significantly lower than that recorded simultaneously in CCKBCs. Firing threshold values are normalized to PVBC firing threshold (CCKBC: 8.67 ± 1.91, *n* = 9 dual recordings; Paired Sample Wilcoxon Signed Rank Test, *p* < 0.01) (S8 Data). (E) Maximum z intensity projection image of the in vitro biocytin-filled interneurons from which data are shown in (B) and (C). Right, images illustrate the presence of calbindin (Calb+) immunopositivity in the PVBC (blue), while epifluorescent images show the expression of enhanced green fluorescent protein (eGFP) in the PVBC and red fluorescent protein (DsRed) in the CCKBC together with the corresponding differential interference contrast images. Scales: 50 μm, insets: 25 μm.

To directly evaluate the recruitment of these GABAergic cells selectively by feedback excitation in the BA, we performed electrophysiological recordings in post hoc–identified interneurons combined with optogenetics. First, we produced triple transgenic mice by crossing VGluT1-cre mice with PV-eGFP x CCK-DsRed double-crossed mice, allowing simultaneous investigations of the 2 interneuron types. Then, an adeno-associated virus (AAV) carrying channelrhodopsin 2 fused to a red fluorescent protein (ChR2-mCherry) expressed in a cre-dependent manner was injected into the BA of these triple transgenic mice, allowing us to selectively excite PNs locally by blue light illumination (Fig 8A). Three to five weeks after injection, acute slices containing the amygdala region were prepared, and simultaneous loose-patch recordings from a PV-containing interneuron and a CCK-expressing interneuron were obtained while the intensity of light illumination was gradually increased, resulting in a cumulative activation of PN populations (Fig 8B). We noticed that spikes could be detected at significantly lower light power in PV-expressing cells in comparison to CCK-containing interneurons (Fig 8C and 8D, S8 Data, S9 Fig, S16 Data). To determine the magnitude of excitatory synaptic inputs necessary to evoke action potentials in these GABAergic cells, the illumination protocol was repeated while the same 2 interneurons were recorded in whole-cell mode (Fig 8B and 8C, S8 Data), allowing the identification of neuron types post hoc (Fig 8E). The results indicated that at the activation level of PNs in which PV-containing interneurons reached their spiking threshold, they received significantly larger evoked excitatory synaptic input than CCKBCs (S9B Fig, S16 Data). This difference was preserved when we separately examined PVBCs (S9B Fig, S16 Data), indicating that PVBCs are driven by lower PN activity levels than CCKBCs. When the PN population activity reached the level required to drive CCKBC firing, PVBCs already discharged multiple spikes (Fig 8B, number of PVBC spikes at CCKBC firing threshold, 4.26 ± 0.21, *n* = 9).

These observations clearly show that the 2 BC types are distinctly recruited by BA PNs, supporting the hypothesis that the functions of PVBCs and CCKBCs are different.

## Discussion

**Fig 9:**
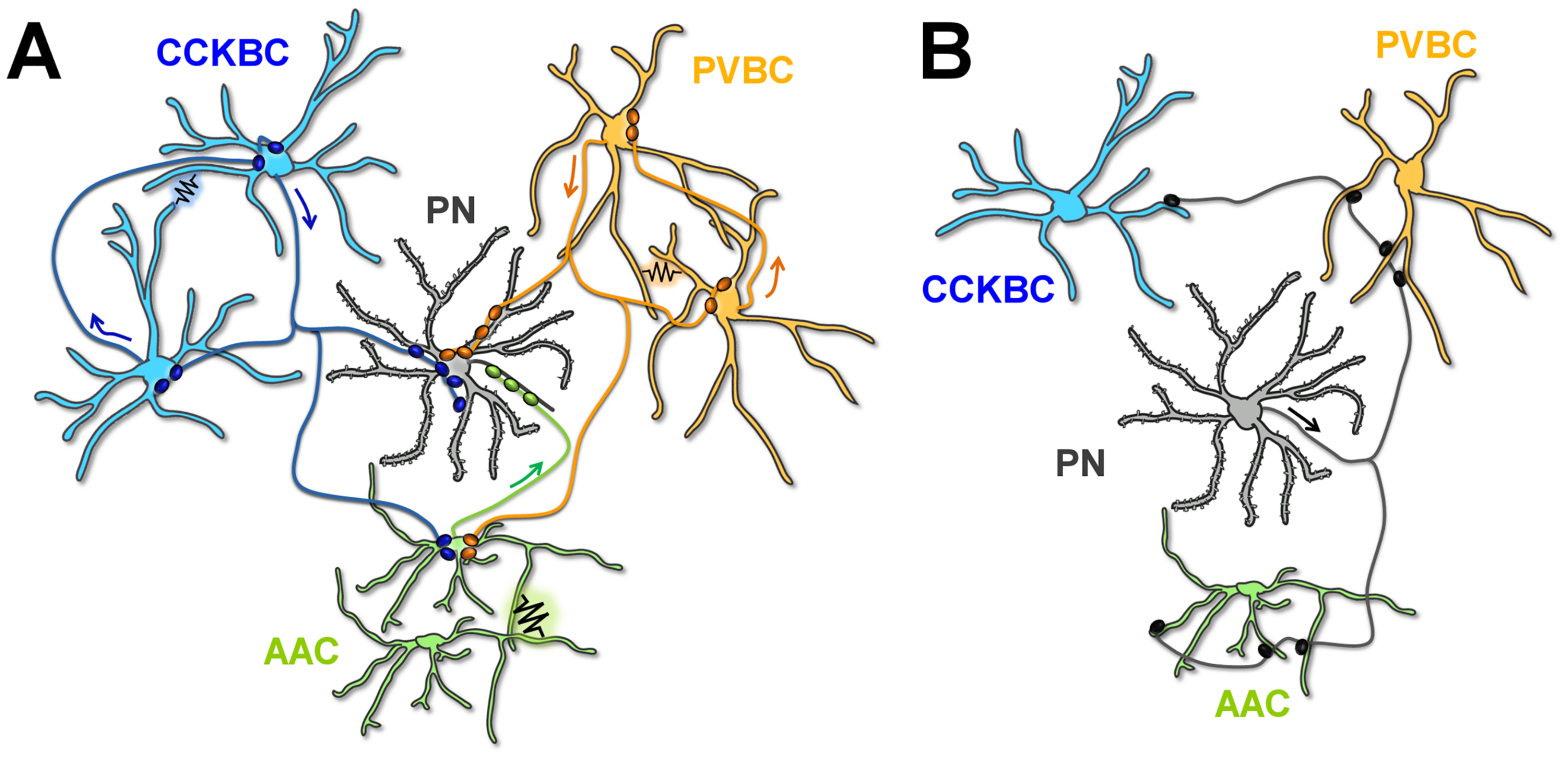
The wiring diagram of the 3 perisomatic region-targeting interneurons and principal neurons (PNs) in the basal amygdala (BA). (A) Connectivity among the distinct interneuron types and from interneurons to the PNs. Both synaptic connections and gap junctions are indicated. Arrows show the direction of action potential spread along the axons. (B) Connectivity from the PNs to interneurons.

Our study is the first to report a detailed wiring diagram of perisomatic inhibitory interneurons in a cortical structure (Fig 9). As found in other cortical areas [20–24], BCs in the BA are mutually interconnected within their own category via both synapses and gap junctions. Our novel findings, that the 2 BC types form parallel inhibitory networks whose distinct feedback recruitment is highly dependent on the local PN activity level, indicate different and independent roles for these GABAergic cell types. Furthermore, the unidirectional connectivity between BCs and AACs may explain the temporal sequence of interneuron firing during synchronous network activities and sensory processing [33, 52–54].

Our conclusion that CCKBCs and PVBCs form 2 parallel networks is based on the results obtained by 2 independent methods. As CCKBCs and PVBCs receive distinct excitation from PNs (present study, [11]), differ in subcortical inputs, and are endowed with specific sets of receptors [8], these results imply that the 2 GABAergic networks should function separately for the most part, probably fulfilling distinct roles in neural computation [55]. This hypothesis is supported by in vivo data showing that the 2 BC types fire distinctly during oscillatory activities in the hippocampus [13], providing temporarily segregated inhibition on the same membrane domain of PNs. Surprisingly, we found that AACs receive inputs from both BC types with high probability and magnitude. Thus, our wiring diagram (Fig 9) suggests that this third perisomatic inhibitory cell type can predominantly, if not exclusively, spike when the 2 BC types are silent. Indeed, hippocampal AACs have been found to be silent during sharp wave-ripple activities when the firing rate of PVBCs is maximal, and in a different phase of theta rhythms in comparison to BCs [9, 53]. Interestingly, AAC firing precedes firing of other interneuron types upon sensory stimuli [33, 54, 56]. These results imply a critical role for AACs at the first stages of sensory processing [57].

The sole communication between AACs can be achieved via electrical coupling (S2 Fig, S10 Data), since these GABAergic interneurons do not form synaptic contacts with each other (present study, [58]). This type of network structure is not unique for AACs in the CNS but has been observed, e.g., among cerebellar Golgi cells whose synchronous activity can be promoted or reduced via gap junctions depending on the input patterns [59, 60]. Thus, synaptic inhibition arriving on the axon initial segments may help the synchronization or desynchronization of the activity in PN ensembles as a function of the gap junction strength among AACs, a signal transfer, which might be subject to plasticity [61].

Previous studies investigating the postsynaptic current characteristics generated by the output synapses of BCs in hippocampal slices suggested that PVBCs and CCKBCs gave rise to synaptic inhibition with different properties [11, 12, 14, 62]. In contrast, our studies in the BA show that the magnitude of the synaptic events originating from the 2 BC types is similar both in young and adult mice (S5 Fig, S12 Data, [37]. The reason for the discrepancy could be 2-fold. First, in distinct cortical structures, the properties of synaptic transmission originating from the 2 BC types might be different. Second, a technical issue might also contribute to the discrepancy. To study synaptic inhibition, experiments are routinely conducted with an intrapipette solution containing high Cl^−^ concentration in postsynaptic neurons, while in our study, we used a low concentration of Cl^−^, similar to physiological conditions [38]. As the intracellular Cl^−^ concentration may alter some of the parameters of GABA_A_ receptor-mediated synaptic transmission [63], the results obtained with distinct Cl^−^ concentrations even at the same synaptic junctions can differ, making it hard to compare the synaptic properties, unless the recording circumstances are as identical as possible. Motivated by this potential concern, we compared the inhibitory efficacy of the 3 perisomatic inhibitory cells using the same conditions. In spite of the differences observed in some of the features in synaptic transmission (present study, [37, 38]), the 3 cell types inhibited PN spiking with equal efficacy. Thus, our results show that the perisomatic region of PNs in the BA receive 3 distinct sources of synaptic inhibition controlling spiking with indistinguishable efficacy.

Earlier anatomical studies obtained in the hippocampus [64, 65] showed that the density of excitatory synapses on the dendrites of PV-expressing interneurons is significantly higher than on the dendrites of CCKBCs. Thus, these 2 GABAergic cell types should be distinctly excited by PN activities, a hypothesis that has been strengthened by slice physiology [11]. Our data confirmed and substantially extended these results with functional implications in the BA by showing that i) lower activity levels of PNs are sufficient to excite PVBCs than CCKBCs; ii) the density of VGluT1-expressing glutamatergic inputs is significantly higher on the dendrites of PVBCs than on the dendrites of CCKBCs and, in parallel, the mEPSC frequency in PVBCs is considerably higher than in CCKBCs; iii) uEPSCs originating from single PNs are larger and faster in PVBCs than in CCKBCs; iv) PVBCs are preferentially innervated by neighboring PNs, while CCKBCs receive excitation from PNs with lower probability but in a distance-independent manner; and v) uEPSC amplitude in PN→CCKBC pairs with single contacts, quantal excitatory events (i.e., mEPSCs) in CCKBCs, and AMPA receptor content at individual synapses along their dendrites show a surprisingly uniform distribution, whereas a high variability characterizes uEPSCs and mEPSC amplitude distributions as well as AMPA receptor content at single clusters in PVBCs. The latter results are specifically interesting because they suggest that the excitatory inputs onto CCKBCs from PNs may have little potency to undergo plastic changes in comparison to those excitatory inputs received by PVBCs. Indeed, long-term potentiation (LTP) or long-term depression (LTD) at the excitatory synapses of hippocampal CCKBCs have not been reported so far [66, 67]. However, these studies as well as others found long-term changes in excitatory transmission in PVBCs [68, 69]. Thus, a network of CCKBCs, due to their nonplastic excitatory inputs, may be excited independently of learning-induced changes, while plastic changes at the excitatory inputs of PVBCs might help memory formation in cortical circuits.

The generality of our present findings is supported by data obtained in the hippocampus, in which the cross-connectivity between the 2 BC types is low (if any) [21], a significantly lower number of excitatory inputs is received by CCKBCs in comparison to PVBCs [64, 65], and PVBCs can be activated more reliably than CCKBCs [11], similarly to those observed in the BA. Although comparable studies have not been performed in neocortical areas, the presence of analogous cell types and the connectivity patterns [47] as well as the documented distance-dependence in the connection probability between pyramidal cells and PVBCs [50, 51, 70] imply that microcircuits in the cerebral cortex may be organized along similar principles as in the BA and hippocampus.

In summary, as the same interneuron types are present in all neocortical structures [49, 71], the synaptic organizing principles revealed here, in the BA, for the perisomatic inhibitory cells, offer a framework for understanding the temporal dynamics of these 3 interneuron types during oscillatory activities and sensory processing. In addition, the 2 BC networks distinctly excited by PNs in a feedback manner may be a general circuit motif in neocortical areas critical for information processing.

## Materials and methods

### Experimental animals and slice preparation

All experiments were approved by the Committee for the Scientific Ethics of Animal Research (22.1/360/3/2011) and were performed according to the guidelines of the institutional ethical code and the Hungarian Act of Animal Care and Experimentation (1998; XXVIII, section 243/1998, renewed in 40/2013) in accordance with the European Directive 86/609/CEE and modified according to the Directives 2010/63/EU. Transgenic or double-transgenic mice of either sex (3 weeks to 10 weeks old) expressing eGFP under the control of the Pvalb promoter (BAC-PV-eGFP, [72]), expressing DsRed under the Cck promoter (BAC-CCK-DsRed, [73]), or expressing both eGFP and DsRed were used in in vitro experiments. For acute slice preparation, mice were deeply anesthetized with isoflurane and decapitated. As described before, the brain was quickly removed and placed into ice-cold solution, containing (in mM): 252 sucrose, 2.5 KCl, 26 NaHCO_3_, 0.5 CaCl_2_, 5 MgCl_2_, 1.25 NaH_2_PO_4_, 10 glucose, bubbled with 95% O_2_/5% CO_2_ (carbogen gas)[37]. Horizontal slices of 200-μm thickness containing the BA were prepared with a Leica VT1000S or VT1200S vibratome and kept in an interface-type holding chamber containing ACSF at 36°C that gradually cooled down to room temperature. ACSF contained the following (in mM): 126 NaCl, 2.5 KCl, 1.25 NaH_2_PO_4_, 2 MgCl_2_, 2 CaCl_2_, 26 NaHCO_3_, and 10 glucose, bubbled with carbogen gas.

### Electrophysiological recordings

After at least 1 hour, incubation slices were transferred to a submerged type recording chamber perfused with 32°C ACSF with approximately 2–2.5 ml/min flow rate. Recordings were performed under visual guidance using differential interference contrast microscopy (Olympus BX61W or Nikon FN-1) using 40x or 16x water dipping objective. Neurons expressing eGFP or DsRed were visualized with the aid of a mercury arc lamp or a monochromator (Till Photonics) and detected with a CCD camera (Hamamatsu Photonics or Andor Zyla). Patch pipettes (4–7 MΩ) for whole-cell recordings were pulled from borosilicate capillaries with inner filament (thin walled, OD 1.5) using a DMZ-Universal Puller (Zeitz Instruments) or using a P1000 pipette puller (Sutter Instruments). For loose-patch recordings 3–5 MΩ pipettes were filled with normal ACSF, and an incomplete seal was formed during the recording with the cell membrane of the targeted neuron in order to monitor spiking activity. In whole-cell paired recordings the patch pipette contained a K-gluconate-based intrapipette solution containing the following (in mM): 110 K-gluconate, 4 NaCl, 2 Mg-ATP, 20 HEPES, 0.1 EGTA, 0.3 GTP (sodium salt), and 10 phosphocreatine adjusted to pH 7.3 using KOH, with an osmolarity of 290 mOsm/L. In interneuron (IN)→PN paired recordings, the presynaptic intrapipette solution additionally contained 0.2% biocytin and 10 mM GABA, and the postsynaptic intrapipette solution additionally contained 100 μM AlexaFluor-488 hydrazide sodium salt (Invitrogen). In PN→IN paired whole-cell recordings, 0.2% biocytin and 10 mM glutamate were added to the presynaptic intrapipette solution, and 0.1 mM spermine and Cascade Blue hydrazide trisodium salt (0.1%), Lucifer Yellow CH potassium salt (0.1%), or Alexa 594 hydrazide sodium salt (100 μM, all from Life Technologies) was added to the postsynaptic intrapipette solution in order to visualize the recorded cells in different colors. In light stimulation experiments, in which only INs were recorded in whole-cell mode, the intrapipette solution contained 0.2% biocytin. In IN→IN paired whole-cell recordings, intrapipette solution for both cells contained the following (in mM): 54 K-gluconate, 4 NaCl, 56 KCl, 20 HEPES, 0.1 EGTA, 10 phosphocreatine, 2 Mg-ATP, 0.3 GTP (sodium salt), 10 mM GABA, and 0.2% biocytin adjusted to pH 7.3 using KOH and with an osmolarity of 290 mOsm/L.

Recordings were performed with a Multiclamp 700B amplifier (Molecular Devices), low-pass filtered at 2 kHz, digitized at 10–50 kHz, and recorded with an in-house data acquisition and stimulus software (Stimulog, courtesy of Prof. Zoltán Nusser, Institute of Experimental Medicine, Hungarian Academy of Sciences, Budapest, Hungary) or Clampex 10.4 (Molecular Devices). Recordings were analyzed with EVAN 1.3 (courtesy of Professor Istvan Mody, Department of Neurology and Physiology, University of California, Los Angeles, CA), Clampfit 10.4 (Molecular Devices), Origin 8.6, or OriginPro 2015. Recordings were not corrected for junction potential. To test the firing characteristics, neurons were injected with 800-ms–long hyperpolarizing and depolarizing square current pulses with increasing amplitudes from −100 to 600 pA. The broad action potential waveform, accommodating firing pattern, and slow after-hyperpolarization were characteristic for PNs. PN identity was further confirmed by the post hoc morphological analysis of their spiny dendrites. For PVBCs and AACs, fast spiking, nonaccommodating firing pattern together with eGFP expression were typical as well as post hoc analysis of their neurochemical marker profile. CCKBCs were characterized by their accommodating regular firing pattern together with strong DsRed expression as well as post hoc analysis of the expression of CB1 at their axon terminals (see below).

For recordings of IPSCs in IN→PN pairs, the presynaptic IN was held near a membrane potential of −65 mV in current-clamp mode and injected by brief square current pulses (2 ms, 1.5–2 nA) to evoke spikes. As done before, the PN was clamped at a holding potential of −40 mV [38]. Series resistance was monitored (range: 8–20 MΩ) and compensated by 65%. To record IPSPs, the presynaptic cell was stimulated in the same way, and the postsynaptic PN was held in current clamp mode at approximately −55 mV. Bridge balance was adjusted throughout the recordings. Kinetic properties of IPSCs and IPSPs were analyzed on the average of 10 to 20 consecutive events. To test the capability of BCs to suppress PN firing, sinusoidal current pulses at theta frequency (3.53 Hz) with peak-to-peak amplitudes of 30 pA and 50 pA were injected into the postsynaptic PN. As done before, the membrane potential of PNs was set (approximately −55 mV) to trigger a spike at the peak of the sinusoidal current pulses with the amplitude of 50 pA but not with 30 pA [38]. This adjustment maintained the membrane potential of PNs near the spiking threshold. One trial consisted of 7 sinusoidal current waves (5 x 50 pA and 2 x 30 pA), repeated 10 to 20 times in each pair. Three action potentials at 30 Hz were evoked in the IN by brief square current pulses (2 ms, 1.5–2 nA) before the fourth sinusoidal current wave (50 pA) in each trial. To assess the reduction in spiking probability, the probability of action potential generation in PNs under control conditions was determined from the average of the responses to 50 pA currents (first, third, fifth, and sixth sinusoidal wave), which was compared with that recorded during the fourth cycle.

In IN→IN pair recordings, presynaptic INs were held in current clamp mode at approximately −65 mV, and 3 to 10 action potentials were evoked by injection of brief square current pulses (2 ms, 1.5–2 nA) at 30 Hz or 40 Hz, while IPSCs in the postsynaptic cell were recorded at the membrane potential of −65 mV. To study the electrical coupling, a hyperpolarizing current of 100 pA or 200 pA in one IN was applied, and the change in the voltage was monitored in the other IN. This experiment was performed bi-directionally. IN pairs were considered electrically coupled when a change in the voltage of one interneuron could also be observed in the other one.

For recordings of excitatory postsynaptic currents/potentials (EPSCs/EPSPs) in PN→IN pairs, postsynaptic INs were recorded at −65 mV, while 3 to 5 brief square current pulses (2 ms, 1.5–2 nA) were applied in the presynaptic PN held at approximately −65 mV in current clamp mode. For constructing the connectivity map, PV-eGFP, CCK-DsRed, or PV-eGFP x CCK-DsRed transgenic mice (P30–35) were injected with 2/5 serotype AAV carrying CaMKII-ChR2-mCherry construct (Penn Vector Core) bilaterally into the BA (anteroposterior: −1.5; mediolateral: 3.3; dorso-ventral: −4.4 mm from bregma, 50–100 nl into each hemisphere). At least 3 weeks after the injection, acute slices of 200-μm thickness were obtained from mice expressing ChR2 in PNs, as described above. For mapping the connection probability between PNs and perisomatic inhibitory INs using light stimulation, an eGFP- or a DsRed-expressing IN was recorded in whole-cell mode, while single PNs were sequentially activated by a blue light spot having a diameter of the soma sized (15–20 μm) for 50 ms (447 nm blue laser, Roithner Laser Technik) using a Digital Mirror Device (DMD)-based pattern illuminator (Mightex Polygon 400). PNs were randomly chosen and the light-evoked action potentials were simultaneously monitored with a pipette containing ACSF in loose-patch mode. The connectivity map was created using the XY coordinates of the recorded cells, and the inter-somatic distance was calculated between the tested presynaptic PNs and the postsynaptic IN. Light stimulation intensity was set individually for each tested presynaptic PN to the minimal intensity value sufficient to evoke action potential(s) (S7 and S10 Figs, S14 Data). We used 50 ms-long light pulses with low stimulus intensity to minimize the light scattering that might activate some neighboring PNs expressing ChR2 and/or their axons. Since the activation threshold of individual ChR2-expressing PNs highly depends on the ChR2 expression level (S7 and S10 Figs, S14 Data), it is expected that in some cases more than one PN is stimulated by the applied method. To overcome this limitation, we peak aligned the light-evoked action potentials in the PN, recorded in loos-patch mode in order to exclude uEPSCs that originated from the light-activated neighboring PNs (S7 Fig, S14 Data). To test a connection, 50–200 sweeps were recorded, and a peri-stimulus histogram was calculated in order to reveal monosynaptic excitatory connections between the PNs and BCs. In some cases, whole-cell recordings were obtained from the presynaptic PN that was proved to be connected to the BC upon light stimulation, and EPSCs in the postsynaptic BC were recorded upon electrical stimulation of the same PN. This approach allowed us to compare the properties of the unitary events evoked by light and electrical stimulation of the presynaptic PNs. Since no significant difference was found between the results obtained by the 2 methods (S7 Fig, S14 Data), the optical stimulation approach was applicable for mapping the connectivity between PNs and BCs (Fig 6 and S8 Fig, S6 and S15 Data). The amplitude of uEPSCs was measured in an individually defined time window calculated from the peristimulus histogram for each pair, including both events and transmission failure. For the potency of the events, uEPSC amplitudes excluding failures were averaged.

For defining the spiking threshold of the INs by population excitation originating from local PNs, double-transgenic mice (PV-eGFP x CCK-DsRed) were crossed with VGluT1-cre homozygote mice, and offsprings were injected with 2/5 serotype AAVs carrying DIO-ChR2-mCherry construct bilaterally into the BA (the same coordinates as described above) in order to obtain ChR2 expression selectively in PNs. At least 3 weeks after the injection, acute slices were prepared from injected mice. To investigate the excitability of the INs, firing threshold in PVBCs and CCKBCs were always simultaneously measured in loose-patch mode, while the whole area of the BA in the slice was stimulated with 10-ms-long blue (447 nm laser) light pulses with gradually increasing intensity (typically delta +5% steps). Spiking threshold was defined as the lowest light intensity (%), in which INs fired single action potentials in response to the light stimulation of a PC population. After determining the firing threshold in loose-patch mode, INs were repatched in whole-cell mode, and the same protocol was performed to measure the magnitude of the light-evoked currents at the intensity values used for loose-patch recordings.

For miniature event analysis, CCKBCs and PVBCs were recorded in whole-cell mode at a holding potential of −65 mV (i.e., at the reversal potential of GABA_A_ receptor-mediated conductance using low intrapipette Cl^−^) in the presence of 0.5 μM tetrodotoxin (TTX, Hello Bio). In these experiments, the intrapipette solution contained (in mM): 110 K-gluconate, 4 NaCl, 2 Mg-ATP, 20 HEPES, 0.1 EGTA, 0.3 GTP (sodium salt), 10 phosphocreatine, 0.2% biocytin, and 0.1 spermine adjusted to pH 7.3 using KOH, with an osmolarity of 290 mOsm/L. During the offline analysis, individual miniature events were detected automatically using an algorithm, and, after visual inspection of each detected event, the peak amplitude and interevent interval of mEPSCs were measured in Clampfit 10.4 (*n* = approximately 300 consecutive events/IN).

### BA delineation in BAC-CCK-DsRed mice

BAC-CCK-DsRed mice (*n* = 2) were intracardially perfused with 4% PFA in 0.1 M phosphate buffer (PB). The brain was removed from the skull and resectioned to 50-μm-thick horizontal slices. To reveal cholinergic fibers, rabbit anti-vesicular acetylcholine transporter (VAChT), 1:1000, Frontier Institute) was used, which was visualized using an AlexaA488-conjugated donkey anti-rabbit antibody (1:500, Jackson). Single-plane fluorescent images were obtained using a Nikon C2 confocal microscope (Plan Fluor 4x objective, N.A. 0.13, xy: 0.8 μm/pixel).

### Immunostainings for identification of the recorded cells

After the recordings, slices were fixed overnight in a solution containing 4% paraformaldehyde (PFA) in 0.1 M phosphate buffer (PB, pH 7.4). In the case of the ultrastructural analysis of the connections with electron microscopy, the fixative solution additionally contained 0.05% glutaradehyde and 15% picric acid. Biocytin-filled recorded cells were visualized either with Cy3, Alexa488 or Alexa647-conjugated streptavidin (1:3000, Molecular Probes or Life Technologies). In those cells in which the fluorescent dyes Alexa488 or Cascade Blue were used for labeling, the signal was amplified by an immunostaining against the fluorophores (rabbit anti-Alexa488 (Invitrogen), rabbit anti-Cascade Blue (Molecular Probes), all 1:1000). Confocal images were taken using a Nikon A1R or C2 microscope (CFI Super Plan Fluor 20X objective, N.A. 0.45; z step size: 1 μm, xy: 0.31 μm/pixel). Using the confocal images, the postsynaptic IN was fully reconstructed in 3D with the Neurolucida 10.53 software (MBF Bioscience), and the putative contact sites from the presynaptic PN were labelled. For the detailed analysis of the putative synaptic sites, higher magnification images were acquired using the same microscopes (CFI Plan Apo VC60X Oil objective, N.A. 1.40; z step size: 0.13 μm, xy: 0.08 μm/pixel). The location analysis of the contact sites was obtained by the Neurolucida Explorer software. Values were corrected for shrinkage and flattening of the tissue (x and y axis: no correction, z axis: 1.7). One PN→PVBC and one PN→CCKBC pair was further processed for electron microscopic analysis to approve the presence of synapses at the contact sites determined by confocal microscopy. Biocytin in PNs was revealed using avidin-biotinylated horseradish peroxidase reaction (ABC; Vector Laboratories) with nickel-intensified 3,3-diaminobenzidine (DAB-Ni), giving a dark reaction product. Rabbit anti-Cascade Blue primary antibody in INs was visualized with biotin-conjugated goat anti-rabbit secondary antibody, with ABC reaction visualized by DAB giving a light brown chromogen. Next, sections were postfixed in 0.5% OsO_4_ with 7% glucose, treated in 10% uranyl acetate, dehydrated in a graded series of ethanol, and embedded in epoxy resin (Durcupan; Sigma). Ultrathin sections of 60-nm thickness were cut, and putative synaptic sites, in which the presynaptic axon formed close appositions with the labelled IN, were examined in serial sections. In both cases, the presence of the synapses could be clearly verified (Fig 7, S7 Data).

Post hoc confirmation of the identity of the INs was performed based on their neurochemical content as follows. After imaging the cell using a confocal laser scanning microscope (Nikon C2 or A1R), only those INs that preserved the axon collaterals were further processed for anatomical identification. For CCKBC identification, an immunostaining using goat anti-CB1 (1:1000; Frontier Institute) was performed, and only those cells in which CB1 receptor expression was found in their boutons were included in the study. To distinguish PVBCs and AACs, an immunostaining against calbindin was performed (rabbit anti-calbindin, 1:3000, Swant, see [34]). PV-containing cells with calbindin expression in the soma and/or axon terminals were considered BCs, whereas those cells that showed no calbindin expression and displayed characteristic cartridges of terminals surrounding putative axon initial segments (AISs) were considered AACs. In some cases, the identity of AACs was strengthened by ankyrin G staining visualizing AISs, as the boutons of biocytin-labeled cells formed close appositions predominantly with ankyrin G-stained profiles [34]. To assess the connection probability between different IN types, we included in the final data set only those recordings in which both cells could be unequivocally identified and had axonal arbor.

### Comparison of excitatory input density of BCs

The estimation of the density of excitatory inputs received by BCs was done as previously described [74]. Biocytin was revealed in in-vitro–filled INs with Alexa488 coupled streptavidin (1:3000, Molecular probes), then, after fixation, slices were resectioned into 40-μm–thin sections. Samples were incubated for 4 nights at 4°C in a solution containing the following primary antibodies and reagents: mouse anti-bassoon (1:1000, Abcam), guinea pig anti-VGluT1 (1:1000, Millipore), 2% normal donkey serum, 0.5% Triton-X 100, and 0.05% Na-azide in 0.1 M PB. Primary antibodies were visualized with Cy3 conjugated donkey anti-mouse and Cy5 conjugated donkey anti-guinea pig secondary antibodies (Jackson Laboratory, 1:500) incubated for 2 hours at room temperature. Sections were then washed and mounted on slides in Vectashield (Vector Laboratories). Confocal images were taken using a Nikon A1R microscope (CFI Plan Apo VC60X Oil objective, NA: 1.40, z step size: 0.13 μm; xy: 0.06 μm/pixel), and analyzed with Neurolucida 10.53 software.

### Super-resolution microscopy on identified interneuron dendrites

To estimate the content of AMPA receptors at synapses of identified IN dendrites, 3D direct stochastic optical reconstruction microscopy (3D-STORM) was performed. For this study, identified CCKBCs and PV-containing INs were intracellularly filled with biocytin as described above. After the electrophysiological experiments, slices were fixed overnight in 4% PFA and resliced to 30-μm–thick sections. Next, an immunostaining to label AMPA receptors was performed as previously described [75]. Briefly, sections were treated for 10 minutes with a solution containing pepsin 1 mg/ml in 0.1 M PB and HCl 0.2 N at 37°C for antigen retrieval and then a solution containing 0.2% Triton-X and NDS 10% together with BSA 2% in 0.1 M PB to prevent unspecific binding of the antibodies. Then, sections were incubated for 5 days at 4°C in a 0.1 M PB solution containing 0.2% Triton-X, 1% NDS, 0.05% Na-azide and the following mixture of primary antibodies: guinea pig anti-pan-AMPAR (Frontier Institute, 1:200) and mouse anti-bassoon (Abcam, 1:3000). The staining was visualized using Alexa488-conjugated streptavidin (Molecular Probes, 1:10000), Alexa647-conjugated donkey anti-guinea pig (Jackson, 1:400) and Cy3-conjugated donkey anti-mouse (Jackson, 1:500).

Sections were prepared for dSTORM imaging as follows. After the staining, they were flat mounted on coverslips and stored dried at 4°C. Samples were embedded in freshly prepared imaging medium immediately before the STORM imaging session [76]. Once a dendritic segment of interest was selected, a high-resolution confocal stack (512x512 pixels, xy: 0.08 μm/pixel, z step size: 0.15 μm) followed by 3D-STORM imaging was performed with an APO TIRF 100x objective, using a Nikon Ti-E inverted microscope equipped with a Nikon C2 confocal scan head, an AndorXion Ultra 897 High Speed EMCCD camera, and a Nikon N-STORM system.

For STORM imaging, a 300-mW laser was used (VFL-P-300-647, MPB Communications). The imaging process for each sample consisted of 5000 cycles of the reporter Alexa647 activation at maximum laser intensity of 30-ms–long frames, with a continuous low-intensity illumination using the 405-nm laser to enhance the activation. To minimize out-of-focus background and focus drift during imaging, all samples were bleached similarly using the 488-nm, 568-nm and 647-nm–laser lines in all the z depth of the sample, and TIRF illumination angle and a Perfect Focus System were applied.

Localization points (LPs) were collected within 600 nm centered in a focal plane contained in a 1–3 μm range from the surface. The resulting coordinates were acquired using the N-STORM module in the NIS-Elements software, setting the intensity height detection range of the bright points from 1000 to 20000 and the CCD baseline to 100.

Confocal stacks from the imaged areas were deconvolved using Huygens software (SVI), and transformed in ImageJ software. The manual alignment of deconvolved confocal and STORM images, as well as the analysis of the number, 2D Convex Hull area and density of LPs was performed using VividSTORM software [76]. To quantify the number of LPs, a region of interest (ROI) was manually drawn around those puncta observed along the dendrite in single focal planes restricted to the central 300 nm, which contained bassoon in close apposition. Puncta depicting AMPA receptors not apposing bassoon were also analyzed (11 out of 30 for CCKBCs, 13 out of 53 for PV-expressing interneurons), and, as there were no significant differences in the number of LPs compared with the bassoon-apposing puncta (*p* = 0.16 for PV-containing interneurons and *p* = 0.70 for CCKBCs), data were pooled. To avoid biasing the analysis, all images were processed equally, and a normalizing factor was introduced in the number of LPs to account for possible technical differences in the staining or imaging sessions. The normalizing factor was obtained as follows: a cluster analysis was performed in a large area immediately surrounding the analyzed dendrite, using the same parameters used for the analysis of the ROIs. The mean number of LPs from all the automatically identified clusters was calculated for every analyzed image and scored based on the averages obtained in all images. The number of LPs in the ROIs was normalized by dividing the raw data obtained for interneurons by the normalizing factor. In addition, to avoid introducing errors derived from the size/shape of the clusters, analyzed puncta fulfilled 2 additional criteria: (1) normalized number of LPs higher than 8, and (2) puncta area larger than 0.07 μm^2^.

### Morphological investigation of IN→IN connectivity

To investigate the connectivity among INs, CCK-DsRed mice (*n* = 2) and wild-type mice (*n* = 2) were transcardially perfused with 4% PFA in 0.1 M PB, and areas containing the amygdala region were sectioned into 50-μm–thin slices, then freeze thawed above liquid nitrogen in 30% sucrose solution. To label connections between CCKBCs and PV-expressing cells, samples from CCK-DsRed mice were incubated for 6 nights at 4°C in the following primary antibody mixture: mouse anti-gephyrin (Synaptic Systems, 1:1000), rabbit anti-parvalbumin (Swant, 1:10.000), guinea pig anti-CB1 (Frontiers Institute, 1:1000), 2% normal donkey serum, and 0.05% Na-azide in 0.1 M PB. Primary antibodies were visualized with the mixture of the following secondary antibodies: Alexa488 conjugated donkey anti-mouse (Molecular Probes, 1:500), Alexa647 conjugated donkey anti-rabbit and Dylight405 conjugated donkey anti-guinea pig (both Jackson Laboratory, 1:500) incubated 1 night at 4°C. To label PV→PV cell connections, samples from wild-type mice were used, and besides labeling gephyrin and parvalbumin as described above, guinea pig anti-VGAT (Frontiers Institute, 1:1000) and goat anti-GAD65/67 (pan-GAD, Frontiers Institute, 1:500) primary antibodies were also applied that were visualized with Cy3 conjugated donkey anti-guinea pig and Cy3 conjugated donkey anti-goat secondary antibodies. Sections were then washed and mounted on slides in Vectashield. Confocal images were taken using a Nikon A1R microscope (CFI Plan Apo VC60X Oil objective, NA: 1.40, z step size: 0.13 μm; xy: 0.06 μm/pixel), and analyzed with Neurolucida 10.53 software. Appositions were only identified as contacts if the postsynaptic anchoring protein of GABA_A_ receptors, gephyrin, was localized at the side of the immunolabeled terminal, which faced the somatic membrane, otherwise the terminals were regarded to form contacts on neighboring structures. In case of PV-immunoreactive terminals on PV-immunostained somata, the presence of VGAT and GAD65/67 immunolabeling identified the PV-containing structure as an axon terminal. CCKBC somata were visualized by the expression of the genetically encoded DsRed fluorescent protein.

### Statistical analysis

In the case of data with nonnormal distribution according to the Shapiro-Wilk test, the Mann–Whitney *U* test, Wilcoxon Signed Rank test, Kolmogorov-Smirnov test, and Kruskal–Wallis ANOVA were used for analysis of the data. To correlate variables from normal distributions, the Pearson’s correlation coefficient was used. All statistics were performed using Origin 8.6 or OriginPro 2015. Data are presented as mean ± SEM.

## Supporting information

S1 Fig Properties of synaptic connections between perisomatic inhibitory cells in the BA. (A) Interneuron→interneuron whole-cell paired recordings were done in horizontal amygdalar slices prepared from PV-eGFP x CCK-DsRed double transgenic mice. (Ce, central amygdala; BA, basal amygdala; Pir, piriform cortex) (B, C) Comparison of the basic properties of IPSCs originating from CCKBCs and PVBCs, including the synaptic failure rate (Kruskal-Wallis ANOVA p < 0.001, CCKBC→CCKBC: 0.60 ± 0.09, n = 11; CCKBC→AAC: 0.50 ± 0.09, n = 9; PVBC→PVBC: 0.10 ± 0.05, n = 6; PVBC→AAC: 0.11 ± 0.05, n = 6), potency (Kruskal-Wallis ANOVA p = 0.7, CCKBC→CCKBC: 29.9 ± 6.7 pA, n = 11; CCKBC→AAC: 43.2 ± 17.2 pA, n = 9; PVBC→PVBC: 34.1 ± 8.9 pA, n = 6; PVBC→AAC: 22.8 ± 4.2 pA, n = 6), latency (Kruskal-Wallis ANOVA p < 0.001, CCKBC→CCKBC: 1.64 ± 0.17 ms, n = 10; CCKBC→AAC: 1.38 ± 0.05 ms, n = 9; PVBC→PVBC: 0.73 ± 0.05 ms, n = 6; PVBC→AAC: 0.90 ± 0.13 ms, n = 6), rise time 10–90% (Kruskal-Wallis ANOVA p = 0.009, CCKBC→CCKBC: 1.00 ± 0.19 ms, n = 10; CCKBC→AAC: 0.54 ± 0.05 ms, n = 9; PVBC→PVBC: 0.71 ± 0.08 ms, n = 6; PVBC→AAC: 0.61 ± 0.07 ms, n = 6), and decay time constant (Kruskal-Wallis ANOVA p = 0.047, CCKBC→CCKBC: 3.8 ± 0.4 ms, n = 10; CCKBC→AAC: 2.5 ± 0.08 ms, n = 9; PVBC→PVBC: 3.1 ± 0.6 ms, n = 4; PVBC→AAC: 2.9 ± 0.7 ms, n = 6)(S9 Data). Mann-Whitney U test: *p < 0.05; **p < 0.01. Each data point on the plots represents an average obtained in a pair recording, lines represent means.
(TIF)

S2 Fig Electrical coupling between perisomatic inhibitory cells in the BA. (A) Diagram and representative traces of two electrically coupled AACs. Representative traces showing the voltage response to a -100 pA hyperpolarizing current injected in AAC1, and monitored simultaneously in AAC1 and AAC2. Scale in AAC1: y: 5 mV, x: 100 ms; AAC2: y: 0.2 mV, x: 100 ms. (B) Electrical coupling efficacy between AAC→AAC, calculated as the % of voltage change observed in the AAC2 compared to the AAC1 in which the current was injected (1.05 ± 0.18%, n = 10)(S10 Data). Line represents mean. (C) Electrical coupling probability matrix of different interneuron→interneuron pairs obtained by dual recordings. In parentheses the number of coupled cells/ the number of dual recordings tested for electrical coupling are shown.
(JPG)

S3 Fig Quantification of somatic CB1-expressing and PV-containing inhibitory inputs on CCKBCs, PVBCs and AACs using multicolor fluorescent immunostainings against gephyrin, CB1, PV and VGAT+panGAD. Appositions were only identified as contacts (arrows) if the postsynaptic scaffolding protein gephyrin was localized on the side of the immunolabeled terminal which faced the somatic membrane, otherwise the terminals were regarded to form contacts on neighboring structures (arrowheads). Calbindin (Calb) immunostaining was used to separate PVBCs and AACs (see Vereczki et al., 2016 [34]). Scale: 5 μm.
(JPG)

S4 Fig Properties of the output synapses of perisomatic region-targeting interneurons in the BA. Comparison of the basic synaptic properties of IPSCs originating from CCKBCs and PVBCs. (A) Failure rate: 0.21 ± 0.07, n = 13 for CCKBC→PN pairs; 0.01 ± 0.01, n = 13 for PVBC→PN pairs; (B) IPSC latency: 1.32 ± 0.09 ms, n = 12 for CCKBC→PN pairs, 0.69 ± 0.03 ms, n = 13 for PVBC→PN pairs; (C) IPSC potency: 86.93 ± 17.32 pA, n = 13 for CCKBC→PN pairs, 150.86 ± 30.08 pA, n = 13 for PVBC→PN pairs; (D) IPSC rise time 10–90%: 1.38 ± 0.15 ms, n = 12 for CCKBC→PN pairs, 0.53 ± 0.01 ms, n = 13 for PVBC→PN pairs; (E) IPSC decay tau: 3.77 ± 0.26 ms, n = 10 for CCKBC→PN pairs, 3.33 ± 0.15 ms, n = 10 for PVBC→PN pairs; (F) IPSC p3/p1: 1.06 ± 0.09 ms, n = 13 for CCKBC→PN pairs, 0.7 ± 0.04 ms, n = 13 for PVBC→PN pairs, Mann-Whitney U test, * p < 0.05. The underlying data are shown in S11 Data. Each data point on the plots represents an averageobtained in a pair recording, lines represent mean.
(TIF)

S5 Fig Comparison of the properties of synaptic transmission obtained in amygdala slices prepared from young (P18-24) and adult (P>45) mice. No significant difference was observed in the peak amplitude (A), 3 IPSP integral (B) and inhibitory efficacy (C) recorded in CCKBC→PN (blue) and PVBC→PN (orange) pairs in the two age groups (adult data, present study; young data, Veres et al., 2017 [37]). The data are available in S12 Data. Each data point on the plots represents an average obtained in a pair recording, lines represent means. p values are the results of statistical comparison using two sample t test.
(TIF)

S6 Fig Connection probability and the basic properties of unitary EPSCs recorded in CCKBCs and PVBCs are different. (A) Comparison of the connection probability between the two BC types shows that local PNs target CCKBCs with a relatively low likelihood in comparison to PVBCs. Data obtained in paired recordings and with light stimulation are pooled (see S7 Fig). Number of connections shown in gray indicates where no detectable EPSCs could be observed in the postsynaptic interneurons in response to action potentials evoked in the presynaptic PNs in a time window of less than 4 ms. Monosynaptic connections between the PNs and interneurons are shown in color. (CCKBC and PVBC in blue and orange, respectively). The number of tested pairs is shown. The potency (B)(CCKBC: 21.5 ± 1.65 pA, PVBC: 94.25 ± 15.08 pA) and the peak amplitude (C) of the unitary EPSCs in CCKBCs are significantly smaller than those recorded in PVBCs (CCKBC: 10.16 ± 2.02 pA, PVBC: 81.78 ± 15.68 pA). (D) The failure rate of the unitary EPSCs in CCKBCs are significantly larger than those recorded in PVBCs (CCKBC: 0.58 ± 0.05, PVBC: 0.25 ± 0.04). The rise time 10–90% (E)(CCKBC: 0.77 ± 0.07 ms, PVBC: 0.4 ± 0.03 ms), the latency (F)(CCKBC: 1.75 ± 0.09 ms, PVBC: 0.92 ± 0.07 ms) and the decay tau (G)(CCKBC: 3 ± 0.23 ms, PVBC: 1.41 ± 0.1 ms) of the unitary EPSCs in CCKBCs are also significantly different than those recorded in PVBCs Mean ± SEM. Mann-Whitney U test, ***p < 0.001. The underlying data are available in S13 Data.
(TIF)

S7 Fig Basic properties of the unitary EPSCs in CCKBCs (blue) and PVBCs (orange) evoked by light stimulation of single PNs and by electrical stimulation of the same PNs in whole-cell mode are not different. (A) Low magnification confocal image shows the ChR2-mCherry expression site in the BA after AAV injection. Scale: 0.5 mm. (Pir.ctx, piriform cortex; Ce, central amygdala) (B) Schematic illustration of the experiment design to record unitary EPSCs using blue light stimulation. (C) Representative traces showing light evoked action potentials (APs) in a single PN recorded in loose-patch mode, a potential synaptic partner to a CCKBC recorded in whole-cell voltage clamp mode (left). Peak alignment of the light evoked APs revealed a monosynaptic excitatory connection to the postsynaptic CCKBC (middle). Whole-cell paired recording of the same monosynaptically coupled PN→CCKBC pair shows similar characteristics of the EPSCs compared with the light stimulation method (right). n = 50 traces; Scales: y: 50 pA/25 pA, loose-patch/whole-cell; y: 50 mV/25 pA whole-cell/whole-cell; x (left): 10 ms, x (middle, right): 2 ms. (D) Representative traces showing light evoked APs in a single PN recorded in loose-patch mode, a potential synaptic partner to a PVBC recorded in whole-cell voltage clamp mode (left). Peak alignment of the light evoked APs revealed a monosynaptic excitatory connection to the postsynaptic PVBC (middle). Whole-cell paired recording of the same PN→PVBC pair shows similar characteristics of EPSCs compared to the light evoked events (right). n = 50 traces; Scales: y: 50 pA/25 pA, loose-patch/whole-cell; y: 50 mV/25 pA whole-cell/whole-cell; x (left): 10 ms, x (middle, right): 2 ms. Comparison between the light stimulation method and the paired whole-cell recording shows no significant difference in the potency (E)(CCKBC: ChR2 stim: 18.52 ± 1.43 pA, Pair. rec.: 17.23 ± 1.62 pA; Paired Sample Wilcoxon Signed Rank Test, p = 0.53; PVBC: ChR2stim: 82.39 ± 13.44 pA, Pair. rec.: 82.51 ± 14.02 pA; Paired Sample Wilcoxon Signed Rank Test, p = 0.75), in the peak amplitude (F)(CCKBC: ChR2 stim: 6.24 ± 1.23 pA, Pair. rec.: 6.42 ± 1.51 pA; Paired Sample Wilcoxon Signed Rank Test, p = 1; PVBC: ChR2 stim: 70.66 ± 14.39 pA, Pair. rec.: 67.9 ± 14.38 pA; Paired Sample Wilcoxon Signed Rank Test, p = 0.2), (G)(CCKBC: ChR2 stim: 0.65 ± 0.07, Pair. rec.: 0.63± 0.08; Paired Sample Wilcoxon Signed Rank Test, p = 1; PVBC: ChR2 stim: 0.22 ± 0.05, Pair. rec.: 0.26 ± 0.05; Paired Sample Wilcoxon Signed Rank Test, p = 0.06), in the rise time 10–90% (H)(CCKBC: ChR2 stim: 0.74 ± 0.09 ms, Pair. rec.: 0.83 ± 0.06 ms; Paired Sample Wilcoxon Signed Rank Test, p = 0.06; PVBC: ChR2 stim: 0.39 ± 0.04 ms, Pair. rec.: 0.4 ± 0.04 ms; Paired Sample Wilcoxon Signed Rank Test, p = 0.97), in the decay tau (I)(CCKBC: ChR2 stim: 2.76 ± 0.23 ms, Pair. rec.: 2.99 ± 0.34 ms; Paired Sample Wilcoxon Signed Rank Test, p = 0.67; PVBC: ChR2 stim: 1.57 ± 0.21 ms, Pair. rec.: 1.41 ± 0.15 ms; Paired Sample Wilcoxon Signed Rank Test, p = 0.2) of unitary EPSCs either in CCKBCs or in PVBCs. (J) The data obtained by light stimulation (ChR2 stim.) normalized to those collected in paired recordings (Pair. rec.) show no major difference. The underlying data are shown in S14 Data. Each pair of data points represents an average obtained in paired recordings. Mean ± SEM is shown in black.
(TIF)

S8 Fig Mapping connectivity between single PNs and a CCKBC using optogenetics. (A) Example experiment (t150422-01), where potential synaptic coupling between thirteen randomly selected PNs and the CCKBC (center, in blue) was tested. Two of the tested PNs innervated the CCKBC (red dots), while no response could be measured by activation of the remaining PNs (gray dots). Concentric circles indicate Δ100 μm distance from the CCKBC in the center. (B) DIC image shows the position and the distance of the recorded PN→CCKBC pair (PN #11). Red circle illustrates the size of the laser illumination spot over a PN soma. Insets, DIC and epifluorescent images taken from the soma of the postsynaptic CCKBC. Scales: 50 μm, insets: 10 μm. (C) Representative traces show recordings from all tested PNs (left panels). Ten consecutive traces (in gray) and averages (in blue). Scales: y: 50 pA/20 pA loose-patch/whole-cell; x: 2 ms. Peri-stimulus histogram was plotted for all tested PNs to reveal a monosynaptic connection between the PNs and the CCKBC (right panels). Arrows (PN #11, PN #13) indicate two monosynaptic connections out of thirteen PNs tested. The data are shown in S15 Data.
(TIF)

S9 Fig PV-expressing interneurons discharge action potentials at lower levels of PN activation than CCKBCs. (A) Comparison of the firing threshold in PV-expressing interneurons (PVeGFP+) and CCKBCs upon the light stimulation of ChR2-expressing PNs in the BA. (Left) PV-expressing cells at a population level have a significantly lower activation threshold compared to the simultaneously recorded CCKBCs (PVeGFP+: 9.55 ± 2.55%, CCKBC: 36 ± 5.43%; Paired Sample Wilcoxon Signed Rank Test). (Right) PVBCs identified *post hoc* by the calbindin content showed significantly lower spiking threshold compared to the simultaneously recorded CCKBCs. (PVBC: 3.47 ± 0.5%; CCKBC: 26.11 ± 6%, Sample Wilcoxon Signed Rank Test). (B) (Left) At the firing threshold PVeGFP+ interneurons received significantly larger light evoked EPSCs (both peak amplitude and area) compared to CCKBCs tested with the same stimulation protocol as in panel (A) (Peak amplitude, upper row): PVeGFP+: 754.9 ± 98.1 pA, CCKBC: 275.1 ± 53.3 pA; Mann-Whitney U test)(Area, lower row): PVeGFP+: 8.15 ± 1.78 nA*ms, CCKBC: 2.96 ± 0.59 nA*ms; Mann-Whitney U test). (Right) PVBCs displayed at their spiking threshold significantly larger EPSC amplitude and area compared to CCKBCs (Peak amplitude, upper row): PVBC: 914.1 ± 91.3 pA, CCKBC: 210.1 ± 54.7pA; Mann-Whitney U test)(Area, lower row): PVBC: 9.51 ± 2.53 nA*ms, CCKBC: 2.07 ± 0.45 nA*ms; Mann-Whitney U test). ***p < 0.001, **p < 0.01, *p < 0.05. Mean ± SEM are shown in black. The Data are available in S16 Data. In B, results of whole cell recordings obtained onlyfor single interneurons were also included.
(TIF)

S10 Fig Illustration of spatial stimulation profile of a ChR2-expressing PN in response to sub-, near-, and over-threshold stimulation in the basal amygdala. (A) Schematic illustration of the stimulation pattern. Squares were illuminated with 447 nm laser light sequentially in a “scanning”-like manner. (B) DIC image of a ChR2-eYFP-expressing PN in the BA (left), and epifluorescent image of the eYFP expression in the same cell (right). Scale: 10 μm. (C) Example threshold calibration of the ChR2-expressing PN shown in (B). (left) Loose patch recordings, overlaid with DIC image showing the area covered by the illumination pattern, show that at the activation threshold PN fire an action potential in response to light stimulation only in that square, which fully cover its somatic region. (right) Light evoked spatial EPSC profile of the same cell recorded in whole-cell voltage-clamp mode indicates that the largest response could be measured in the same square where PN fired an action potential in response to light stimulation. Scale: 50 μm. (D) Spatial profiles of the same PN activation (number of light evoked AP(s)/square) (left), and light evoked EPSC amplitudes (pA) (right) using sub-threshold stimulation parameters (light pulse length: 1 ms, light intensity 100%). With sub-threshold stimulation parameters no light evoked action potential could be detected. (E, left) Increasing the light pulse length (light pulse length: 2 ms, light intensity: 100%) readily activates the same PN. Near-threshold stimulation parameters evoke firing in the PN, if the light beam targets only the square, which covers the somatic region of the PN. (right) Spatial near-threshold stimulation evoked EPSC profile recorded in the PN. (F) Increasing further the stimulation light pulse length (light pulse length: 5 ms, light intensity: 100%) extended the area where the PN could be activated. (left) Targeting the somatic region covering square with over-threshold stimulation parameters evokes multiple spikes in the PN, but stimulating the neighboring area can also activate the PN. (right) Spatial over-threshold stimulation evoked EPSC profile in the PN.
(TIF)

S1 Data Data for Fig 1G and 1H. (XLSX)

S2 Data Data for Fig 2B. (XLSX)

S3 Data Data for Fig 3E, 3F and 3I. (XLSX)

S4 Data Data for Fig 4C. (XLSX)

S5 Data Data for Fig 5B and 5E. (XLSX)

S6 Data Data for Fig 6C, 6E and 6F. (XLSX)

S7 Data Data for Fig 7J and 7M. (XLSX)

S8 Data Data for Fig 8C and 8D. (XLSX)

S9 Data Data for S1B and S1C Fig. (XLSX)

S10 Data Data for S2B Fig. (XLSX)

S11 Data Data for S4A–S4F Fig. (XLSX)

S12 Data Data for S5A–S5C Fig. (XLSX)

S13 Data Data for S6A–S6G Fig. (XLSX)

S14 Data Data for S7E–S7J Fig. (XLSX)

S15 Data Data for S8A and S8C Fig. (XLSX)

S16 Data Data for S9A and S9B Fig. (XLSX)

## Abbreviations

AAC: axo-axonic cells
AMPA: α-amino-3-hydroxy-5-methyl-4-isoxazolepropionic acid
BA: basal amygdala
BAa: anterior part of the basal amygdala
BAp: posterior part of the basal amygdala
BC: basket cell
BLA: basolateral amygdala complex
Calb: calbindin
CB1: cannabinoid receptor type 1
CCK: cholecystokinin
CCKBC: cholecystokinin-expressing basket cells
CCK-DsRed: expressing red fluorescent protein under the control of the Cck promoter
CV: coefficient of variation
DsRed: red fluorescent protein
eGFP: enhanced green fluorescent protein
EPSC: excitatory postsynaptic current
GABA: gamma-aminobutyric acid
GABA_A_: type A GABA receptor
IPSC: inhibitory postsynaptic current
IPSP: inhibitory postsynaptic potential
LP: localization point
LTD: long-term depression
LTP: long-term potentiation
mEPSC: miniature excitatory postsynaptic current
PanGAD: glutamate decarboxylase 65/67
PN: principal neuron
PV: parvalbumin
PVBC: parvalbumin-containing basket cells
PV-eGFP: expressing enhanced green fluorescent protein under the control of the Pvalb promoter
ROI: region of interest
STORM: stochastic optical reconstruction microscopy
TTX: tetrodotoxin
uEPSC: unitary excitatory postsynaptic current
uIPSC: unitary inhibitory postsynaptic current
VAChT: vesicular acetylcholine transporter
VGAT: vesicular GABA transporter
VGluT1: vesicular glutamate transporter 1
