# Supplementary figures and images for: Differential excitatory control of 2 parallel basket cell networks in amygdala microcircuits
Source: PLoS Biol. 2017 May 24;15(5):e2001421. doi: 10.1371/journal.pbio.2001421 (PMC5443504; doi:10.1371/journal.pbio.2001421)

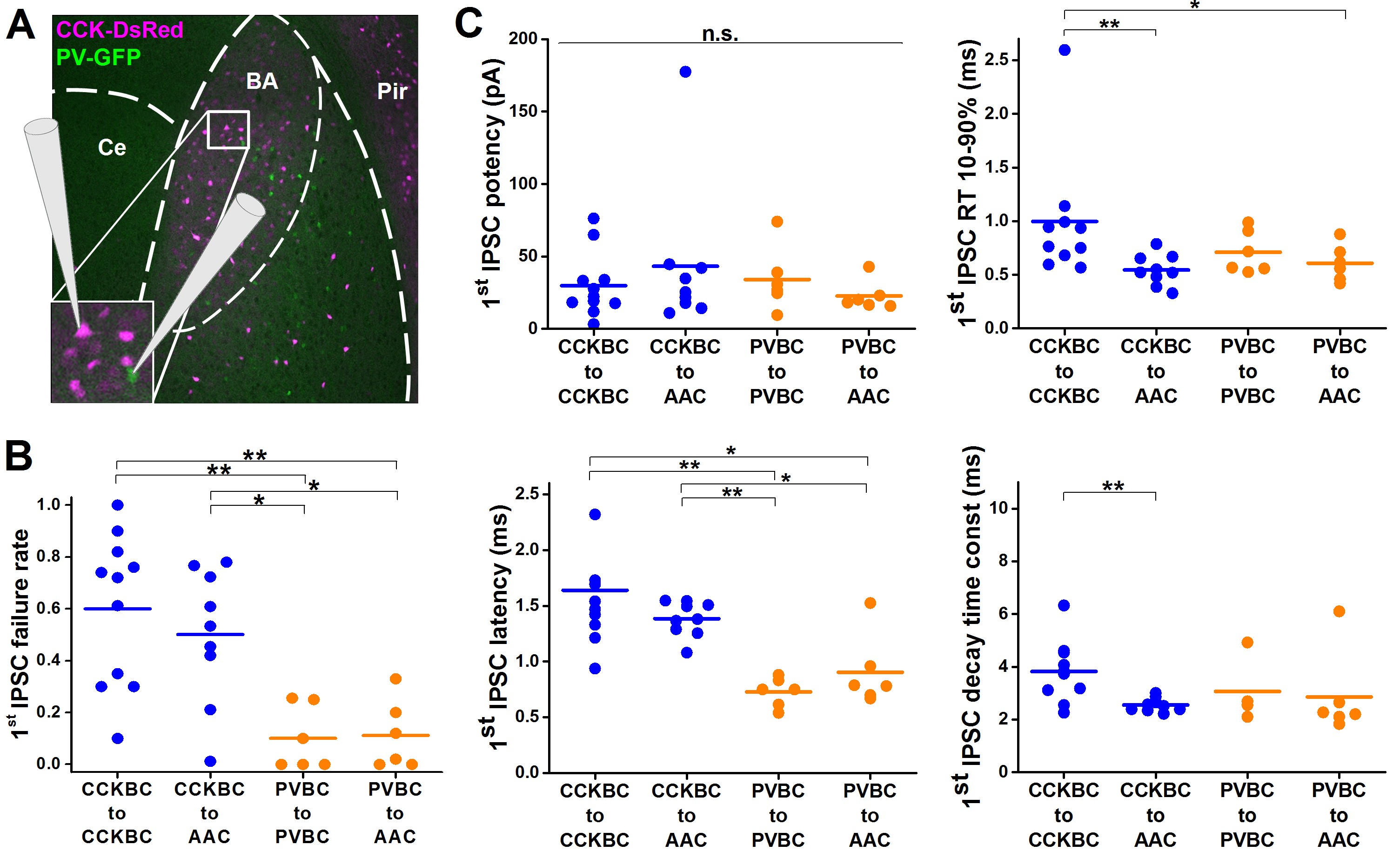

Supplement: S1 Fig — (A) Interneuron→interneuron whole-cell paired recordings were done in horizontal amygdalar slices prepared from PV-eGFP x CCK-DsRed double transgenic mice. (Ce, central amygdala; BA, basal amygdala; Pir, piriform cortex) (B, C) Comparison of the basic properties of IPSCs originating from CCKBCs and PVBCs, including the synaptic failure rate (Kruskal-Wallis ANOVA p < 0.001, CCKBC→CCKBC: 0.60 ± 0.09, n = 11; CCKBC→AAC: 0.50 ± 0.09, n = 9; PVBC→PVBC: 0.10 ± 0.05, n = 6; PVBC→AAC: 0.11 ± 0.05, n = 6), potency (Kruskal-Wallis ANOVA p = 0.7, CCKBC→CCKBC: 29.9 ± 6.7 pA, n = 11; CCKBC→AAC: 43.2 ± 17.2 pA, n = 9; PVBC→PVBC: 34.1 ± 8.9 pA, n = 6; PVBC→AAC: 22.8 ± 4.2 pA, n = 6), latency (Kruskal-Wallis ANOVA p < 0.001, CCKBC→CCKBC: 1.64 ± 0.17 ms, n = 10; CCKBC→AAC: 1.38 ± 0.05 ms, n = 9; PVBC→PVBC: 0.73 ± 0.05 ms, n = 6; PVBC→AAC: 0.90 ± 0.13 ms, n = 6), rise time 10–90% (Kruskal-Wallis ANOVA p = 0.009, CCKBC→CCKBC: 1.00 ± 0.19 ms, n = 10; CCKBC→AAC: 0.54 ± 0.05 ms, n = 9; PVBC→PVBC: 0.71 ± 0.08 ms, n = 6; PVBC→AAC: 0.61 ± 0.07 ms, n = 6), and decay time constant (Kruskal-Wallis ANOVA p = 0.047, CCKBC→CCKBC: 3.8 ± 0.4 ms, n = 10; CCKBC→AAC: 2.5 ± 0.08 ms, n = 9; PVBC→PVBC: 3.1 ± 0.6 ms, n = 4; PVBC→AAC: 2.9 ± 0.7 ms, n = 6)(S9 Data). Mann-Whitney U test: *p < 0.05; **p < 0.01. Each data point on the plots represents an average obtained in a pair recording, lines represent means. (TIF) [file pbio.2001421.s001.tif]

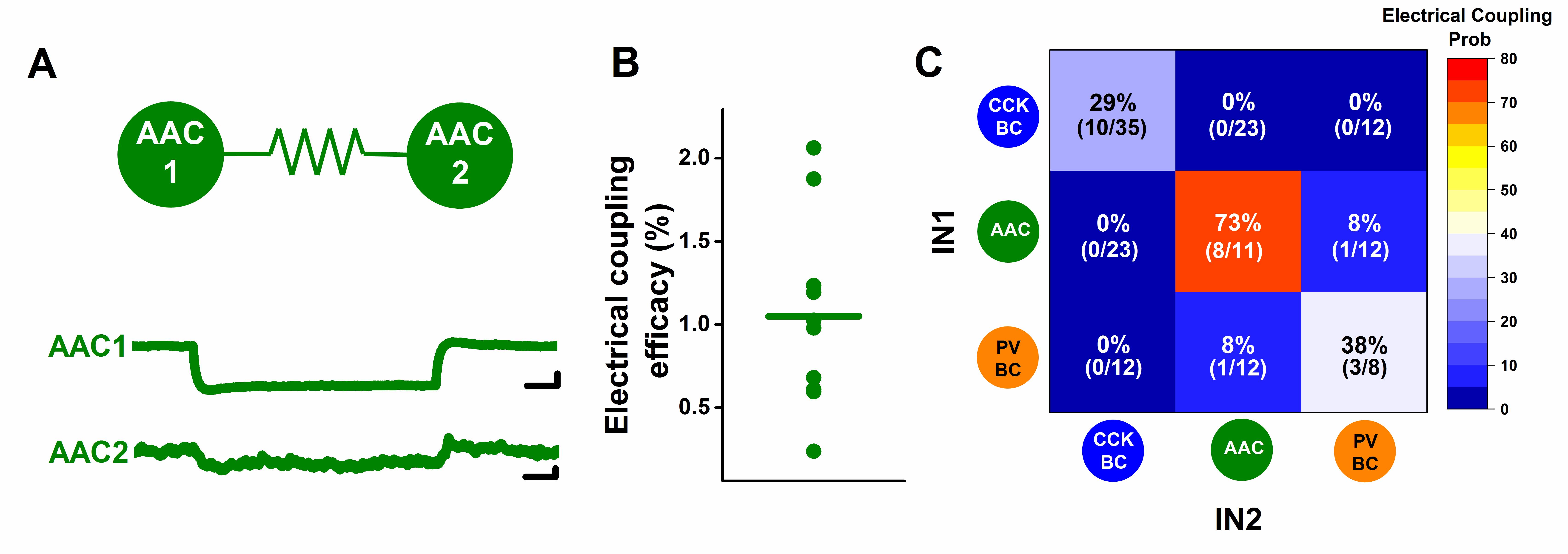

Supplement: S2 Fig — (A) Diagram and representative traces of two electrically coupled AACs. Representative traces showing the voltage response to a -100 pA hyperpolarizing current injected in AAC1, and monitored simultaneously in AAC1 and AAC2. Scale in AAC1: y: 5 mV, x: 100 ms; AAC2: y: 0.2 mV, x: 100 ms. (B) Electrical coupling efficacy between AAC→AAC, calculated as the % of voltage change observed in the AAC2 compared to the AAC1 in which the current was injected (1.05 ± 0.18%, n = 10)(S10 Data). Line represents mean. (C) Electrical coupling probability matrix of different interneuron→interneuron pairs obtained by dual recordings. In parentheses the number of coupled cells/ the number of dual recordings tested for electrical coupling are shown. (JPG) [file pbio.2001421.s002.jpg]

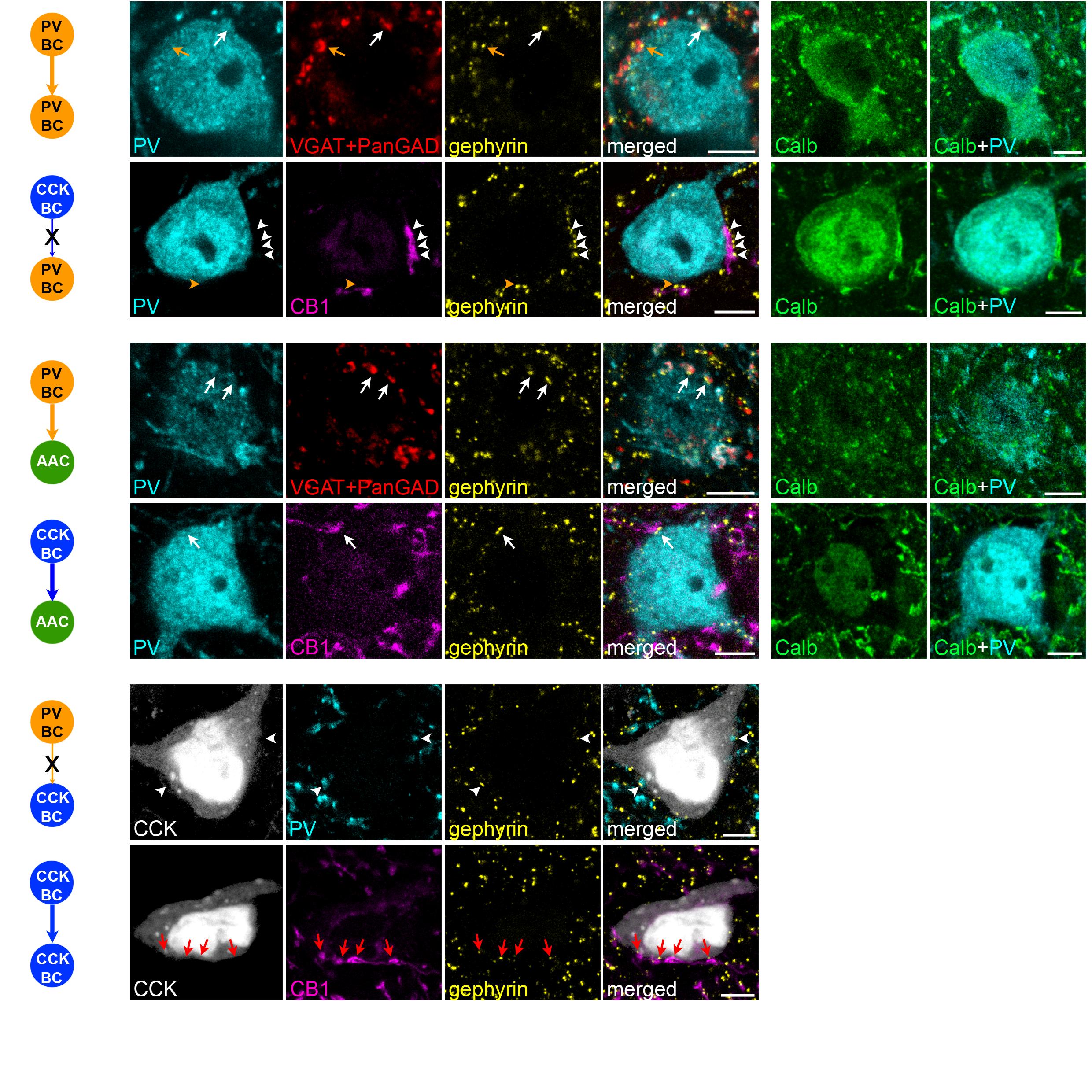

Supplement: S3 Fig — Appositions were only identified as contacts (arrows) if the postsynaptic scaffolding protein gephyrin was localized on the side of the immunolabeled terminal which faced the somatic membrane, otherwise the terminals were regarded to form contacts on neighboring structures (arrowheads). Calbindin (Calb) immunostaining was used to separate PVBCs and AACs (see Vereczki et al., 2016 [34]). Scale: 5 μm. (JPG) [file pbio.2001421.s003.jpg]

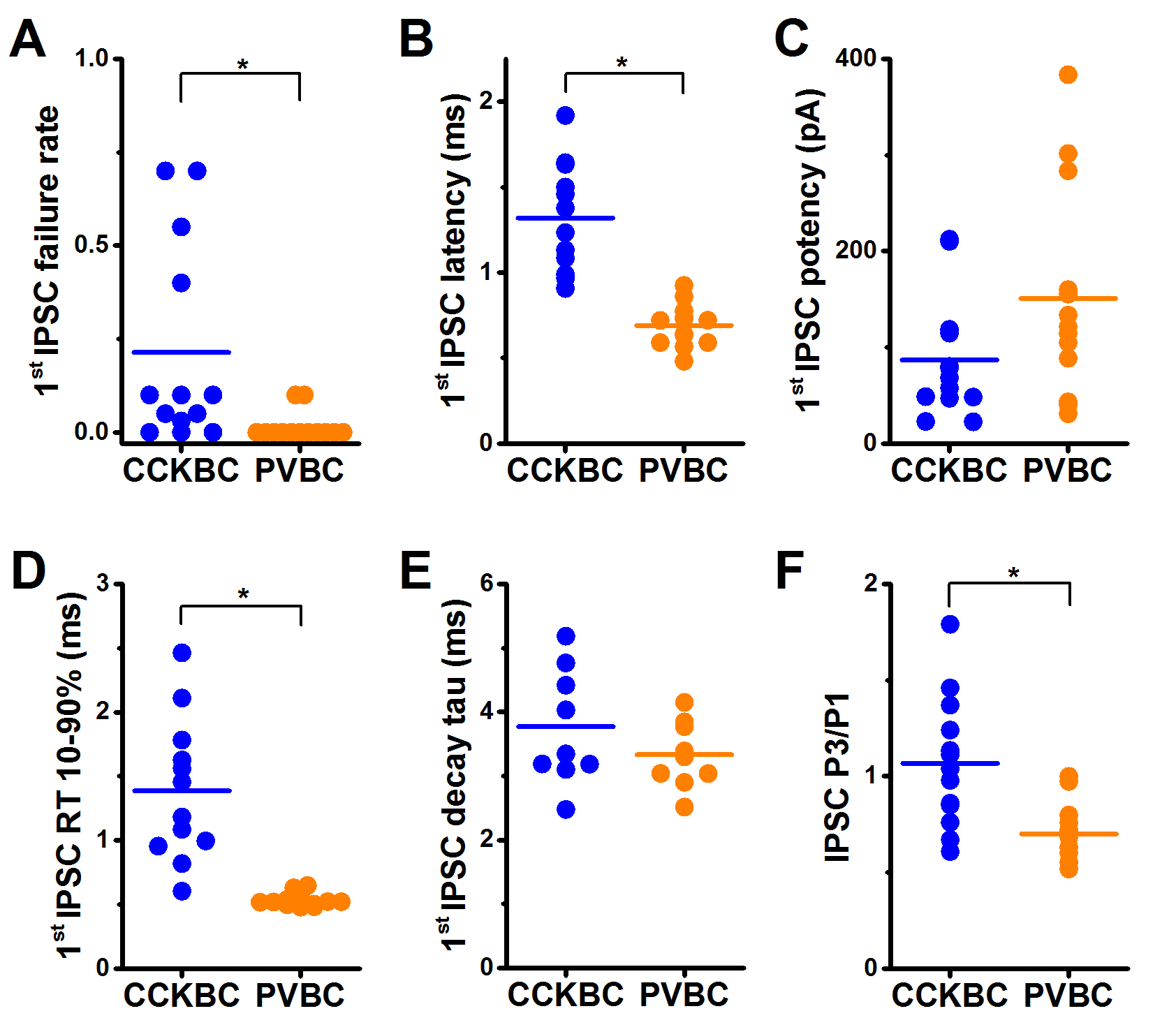

Supplement: S4 Fig — Comparison of the basic synaptic properties of IPSCs originating from CCKBCs and PVBCs. (A) Failure rate: 0.21 ± 0.07, n = 13 for CCKBC→PN pairs; 0.01 ± 0.01, n = 13 for PVBC→PN pairs; (B) IPSC latency: 1.32 ± 0.09 ms, n = 12 for CCKBC→PN pairs, 0.69 ± 0.03 ms, n = 13 for PVBC→PN pairs; (C) IPSC potency: 86.93 ± 17.32 pA, n = 13 for CCKBC→PN pairs, 150.86 ± 30.08 pA, n = 13 for PVBC→PN pairs; (D) IPSC rise time 10–90%: 1.38 ± 0.15 ms, n = 12 for CCKBC→PN pairs, 0.53 ± 0.01 ms, n = 13 for PVBC→PN pairs; (E) IPSC decay tau: 3.77 ± 0.26 ms, n = 10 for CCKBC→PN pairs, 3.33 ± 0.15 ms, n = 10 for PVBC→PN pairs; (F) IPSC p3/p1: 1.06 ± 0.09 ms, n = 13 for CCKBC→PN pairs, 0.7 ± 0.04 ms, n = 13 for PVBC→PN pairs, Mann-Whitney U test, * p < 0.05. The underlying data are shown in S11 Data. Each data point on the plots represents an averageobtained in a pair recording, lines represent mean. (TIF) [file pbio.2001421.s004.tif]

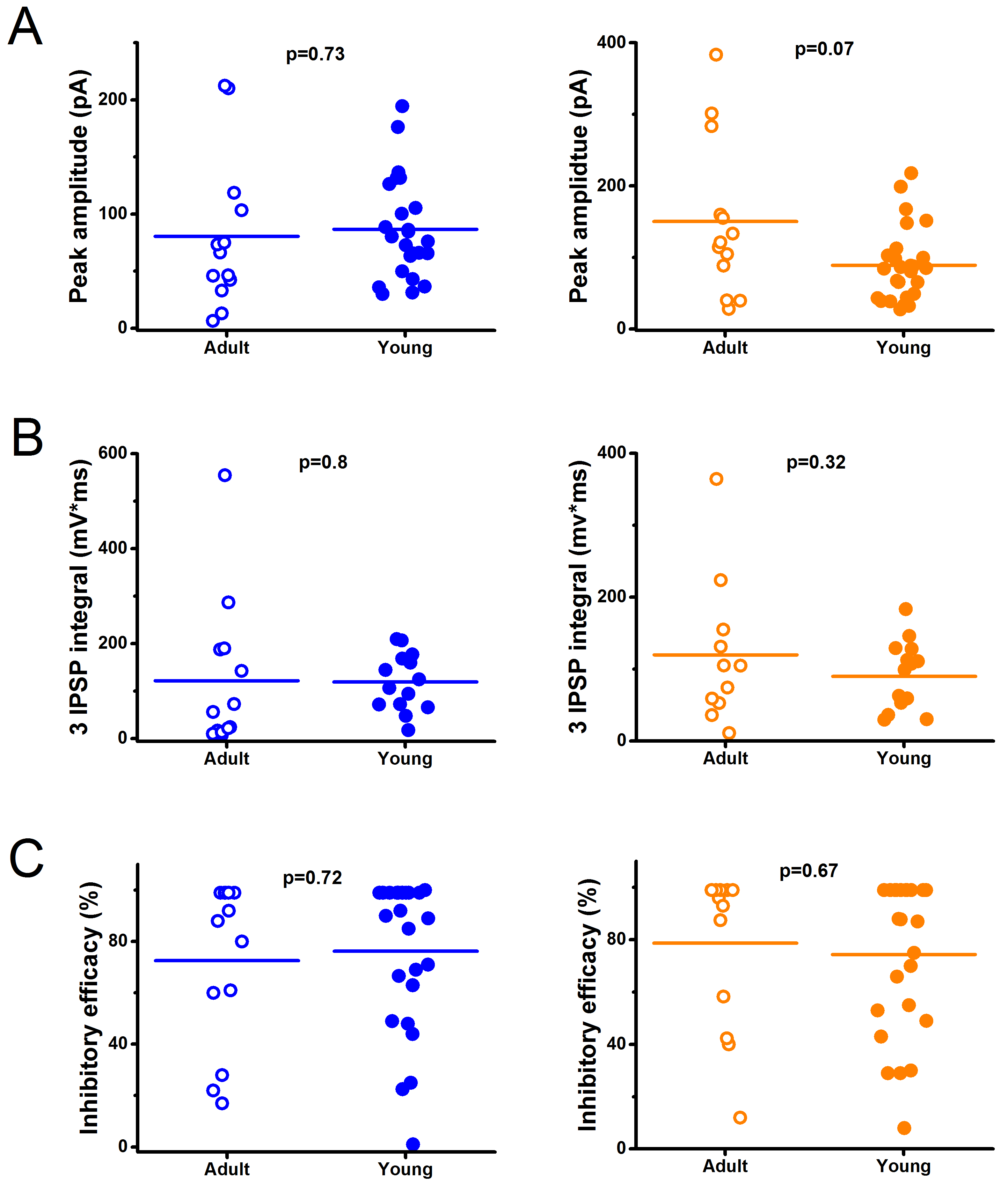

Supplement: S5 Fig — No significant difference was observed in the peak amplitude (A), 3 IPSP integral (B) and inhibitory efficacy (C) recorded in CCKBC→PN (blue) and PVBC→PN (orange) pairs in the two age groups (adult data, present study; young data, Veres et al., 2017 [37]). The data are available in S12 Data. Each data point on the plots represents an average obtained in a pair recording, lines represent means. p values are the results of statistical comparison using two sample t test. (TIF) [file pbio.2001421.s005.tif]

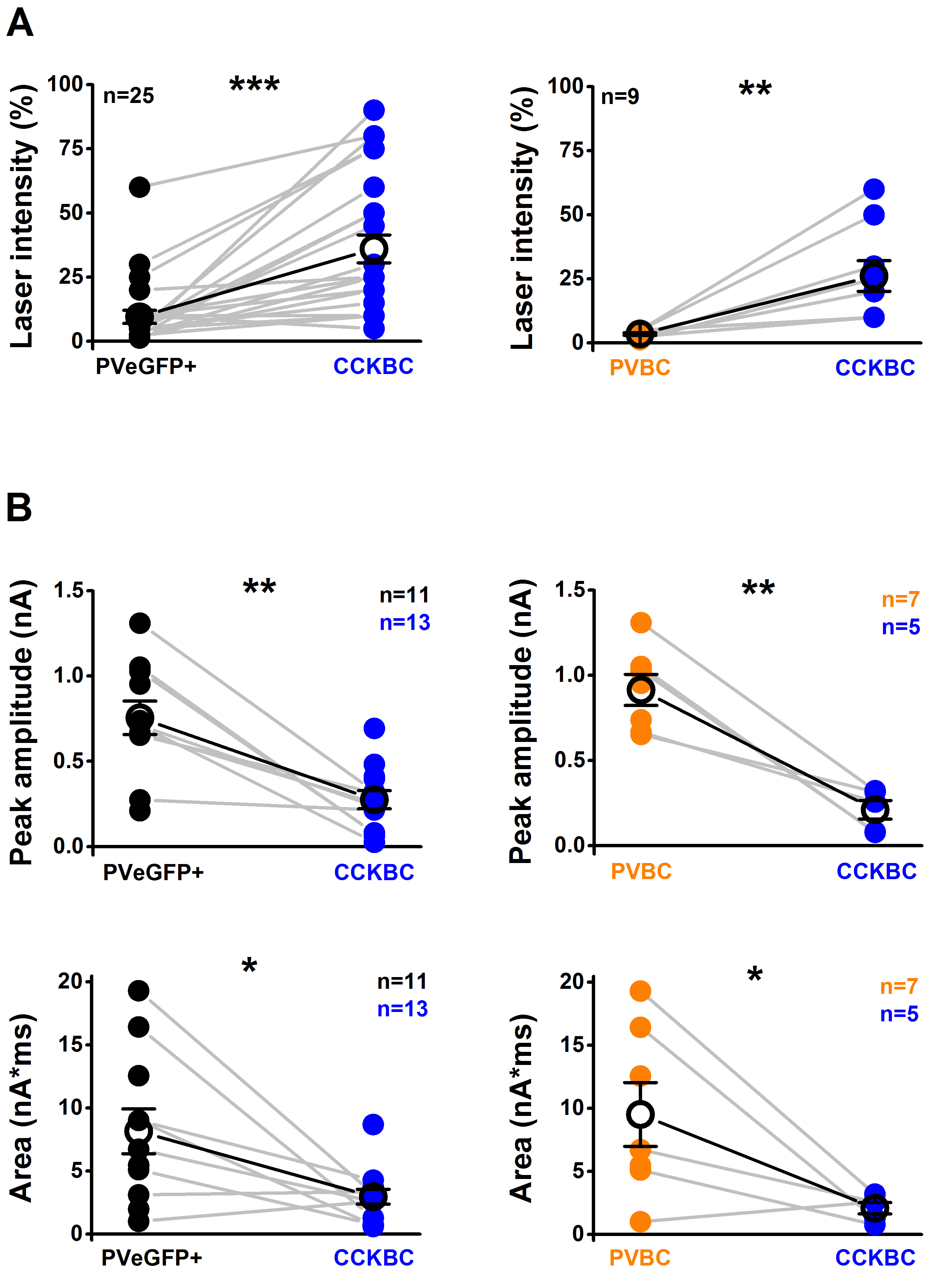

Supplement: S9 Fig — (A) Comparison of the firing threshold in PV-expressing interneurons (PVeGFP+) and CCKBCs upon the light stimulation of ChR2-expressing PNs in the BA. (Left) PV-expressing cells at a population level have a significantly lower activation threshold compared to the simultaneously recorded CCKBCs (PVeGFP+: 9.55 ± 2.55%, CCKBC: 36 ± 5.43%; Paired Sample Wilcoxon Signed Rank Test). (Right) PVBCs identified post hoc by the calbindin content showed significantly lower spiking threshold compared to the simultaneously recorded CCKBCs. (PVBC: 3.47 ± 0.5%; CCKBC: 26.11 ± 6%, Sample Wilcoxon Signed Rank Test). (B) (Left) At the firing threshold PVeGFP+ interneurons received significantly larger light evoked EPSCs (both peak amplitude and area) compared to CCKBCs tested with the same stimulation protocol as in panel (A) (Peak amplitude, upper row): PVeGFP+: 754.9 ± 98.1 pA, CCKBC: 275.1 ± 53.3 pA; Mann-Whitney U test)(Area, lower row): PVeGFP+: 8.15 ± 1.78 nA*ms, CCKBC: 2.96 ± 0.59 nA*ms; Mann-Whitney U test). (Right) PVBCs displayed at their spiking threshold significantly larger EPSC amplitude and area compared to CCKBCs (Peak amplitude, upper row): PVBC: 914.1 ± 91.3 pA, CCKBC: 210.1 ± 54.7pA; Mann-Whitney U test)(Area, lower row): PVBC: 9.51 ± 2.53 nA*ms, CCKBC: 2.07 ± 0.45 nA*ms; Mann-Whitney U test). ***p < 0.001, **p < 0.01, *p < 0.05. Mean ± SEM are shown in black. The Data are available in S16 Data. In B, results of whole cell recordings obtained onlyfor single interneurons were also included. (TIF) [file pbio.2001421.s009.tif]
